# Duration of Untreated Psychosis and Outcomes in First-Episode Psychosis: Systematic Review and Meta-analysis of Early Detection and Intervention Strategies

**DOI:** 10.1093/schbul/sbae017

**Published:** 2024-03-16

**Authors:** Gonzalo Salazar de Pablo, Daniel Guinart, Alvaro Armendariz, Claudia Aymerich, Ana Catalan, Luis Alameda, Maria Rogdaki, Estrella Martinez Baringo, Joan Soler-Vidal, Dominic Oliver, Jose M Rubio, Celso Arango, John M Kane, Paolo Fusar-Poli, Christoph U Correll

**Affiliations:** Department of Child and Adolescent Psychiatry, Institute of Psychiatry, Psychology & Neuroscience, King’s College London, London, UK; Department of Psychosis Studies, Early Psychosis: Interventions and Clinical-detection (EPIC) Lab, Institute of Psychiatry, Psychology & Neuroscience, King’s College London, London, UK; Child and Adolescent Mental Health Services, South London and Maudsley NHS Foundation Trust, London, UK; Department of Child and Adolescent Psychiatry, Institute of Psychiatry and Mental Health, Hospital General Universitario Gregorio Marañón School of Medicine, Universidad Complutense, IiSGM, CIBERSAM, Madrid, Spain; Institut de Salut Mental, Hospital del Mar, Centro de Investigación Biomédica en Red de Salud Mental (CIBERSAM), Barcelona, Spain; Department of Psychiatry, Hospital del Mar Medical Research Institute, Barcelona, Spain; Department of Psychiatry, The Zucker Hillside Hospital, Northwell Health, Glen Oaks, NY, USA; Department of Psychiatry and Molecular Medicine, Zucker School of Medicine at Hofstra/Northwell, Hempstead, NY, USA; Parc Sanitari Sant Joan de Déu, Sant Boi de Llobregat, Spain; Etiopatogenia i Tractament Dels Trastorns Mental Severs (MERITT), Institut de Recerca Sant Joan de Déu, Esplugues de Llobregat, Spain; Psychiatry Department, Basurto University Hospital, Biocruces Bizkaia Health Research Institute, OSI Bilbao-Basurto, Barakaldo, Bizkaia, Spain; Department of Psychosis Studies, Early Psychosis: Interventions and Clinical-detection (EPIC) Lab, Institute of Psychiatry, Psychology & Neuroscience, King’s College London, London, UK; Psychiatry Department, Basurto University Hospital, Biocruces Bizkaia Health Research Institute, OSI Bilbao-Basurto, Barakaldo, Bizkaia, Spain; Department of Psychosis Studies, Institute of Psychiatry, Psychology and Neuroscience, King’s College London, London, UK; TiPP Program Department of Psychiatry, Service of General Psychiatry, Lausanne University Hospital, Lausanne, Switzerland; Department of Psychiatry, Centro Investigación Biomedica en Red de Salud Mental (CIBERSAM), Instituto de Biomedicina de Sevilla (IBIS), Hospital Universitario Virgen del Rocío, University of Sevilla, Sevilla, Spain; Department of Child and Adolescent Psychiatry, Institute of Psychiatry, Psychology & Neuroscience, King’s College London, London, UK; Department of Child and Adolescent Psychiatry, Hospital Sant Joan de Déu de Barcelona, Esplugues de Llobregat, Spain; FIDMAG Germanes Hospitalàries Research Foundation, Barcelona, Spain; Centro de Investigación Biomédica en Red de Salud Mental (CIBERSAM), ISCIII, Barcelona, Spain; Hospital Benito Menni CASM, Hermanas Hospitalarias, Sant Boi de Llobregat, Spain; Department of Psychosis Studies, Early Psychosis: Interventions and Clinical-detection (EPIC) Lab, Institute of Psychiatry, Psychology & Neuroscience, King’s College London, London, UK; Department of Psychiatry, University of Oxford, Oxford, UK; NIHR Oxford Health Biomedical Research Centre, Oxford, UK; OPEN Early Detection Service, Oxford Health NHS Foundation Trust, Oxford, UK; Department of Psychiatry, The Zucker Hillside Hospital, Northwell Health, Glen Oaks, NY, USA; Department of Psychiatry and Molecular Medicine, Zucker School of Medicine at Hofstra/Northwell, Hempstead, NY, USA; Center for Psychiatric Neuroscience, The Feinstein Institutes for Medical Research, Manhasset, NY, USA; Department of Child and Adolescent Psychiatry, Institute of Psychiatry and Mental Health, Hospital General Universitario Gregorio Marañón School of Medicine, Universidad Complutense, IiSGM, CIBERSAM, Madrid, Spain; Department of Psychiatry, The Zucker Hillside Hospital, Northwell Health, Glen Oaks, NY, USA; Department of Psychiatry and Molecular Medicine, Zucker School of Medicine at Hofstra/Northwell, Hempstead, NY, USA; Center for Psychiatric Neuroscience, The Feinstein Institutes for Medical Research, Manhasset, NY, USA; Department of Psychosis Studies, Early Psychosis: Interventions and Clinical-detection (EPIC) Lab, Institute of Psychiatry, Psychology & Neuroscience, King’s College London, London, UK; Department of Brain and Behavioral Sciences, University of Pavia, Pavia, Italy; OASIS Service, South London and Maudsley NHS Foundation Trust, London, UK; Maudsley Biomedical Research Centre, National Institute for Health Research, South London and Maudsley NHS Foundation Trust, London, UK; Department of Psychiatry, The Zucker Hillside Hospital, Northwell Health, Glen Oaks, NY, USA; Department of Psychiatry and Molecular Medicine, Zucker School of Medicine at Hofstra/Northwell, Hempstead, NY, USA; Center for Psychiatric Neuroscience, The Feinstein Institutes for Medical Research, Manhasset, NY, USA; Department of Child and Adolescent Psychiatry, Charité Universitätsmedizin, Berlin, Germany

**Keywords:** duration of untreated psychosis, outcome, early detection, early intervention, meta-analysis

## Abstract

**Background:**

The role of duration of untreated psychosis (DUP) as an early *detection* and *intervention* target to improve outcomes for individuals with first-episode psychosis is unknown.

**Study Design:**

PRISMA/MOOSE-compliant systematic review to identify studies until February 1, 2023, with an intervention and a control group, reporting DUP in both groups. Random effects meta-analysis to evaluate (1) differences in DUP in early detection/intervention services vs the control group, (2) the efficacy of early detection strategies regarding eight real-world outcomes at baseline (service entry), and (3) the efficacy of early intervention strategies on ten real-world outcomes at follow-up. We conducted quality assessment, heterogeneity, publication bias, and meta-regression analyses (PROSPERO: CRD42020163640).

**Study Results:**

From 6229 citations, 33 intervention studies were retrieved. The intervention group achieved a small DUP reduction (Hedges’ *g* = 0.168, 95% CI = 0.055–0.283) vs the control group. The early *detection* group had better functioning levels (*g* = 0.281, 95% CI = 0.073–0.488) at baseline. Both groups did not differ regarding total psychopathology, admission rates, quality of life, positive/negative/depressive symptoms, and employment rates (*P* > .05). Early *interventions* improved quality of life (*g* = 0.600, 95% CI = 0.408–0.791), employment rates (*g* = 0.427, 95% CI = 0.135–0.718), negative symptoms (*g* = 0.417, 95% CI = 0.153–0.682), relapse rates (*g* = 0.364, 95% CI = 0.117–0.612), admissions rates (*g* = 0.335, 95% CI = 0.198–0.468), total psychopathology (*g* = 0.298, 95% CI = 0.014–0.582), depressive symptoms (*g* = 0.268, 95% CI = 0.008–0.528), and functioning (*g* = 0.180, 95% CI = 0.065–0.295) at follow-up but not positive symptoms or remission (*P* > .05).

**Conclusions:**

Comparing interventions targeting DUP and control groups, the impact of early *detection* strategies on DUP and other correlates is limited. However, the impact of early *intervention* was significant regarding relevant outcomes, underscoring the importance of supporting early *intervention* services worldwide.

## Introduction

Schizophrenia is one of the most debilitating and functionally limiting disorders.^1,2^ To ameliorate poor outcomes of psychosis during its early clinical stages,^3^ early *detection* and early *intervention* have the potential to impact the critical period before and after the first episode of psychosis (FEP).^4,5^ Early *detection* focuses on the detection of early signs and symptoms and is based on community awareness^6^ and outreach efforts^7^ to reduce delays in access to care, which are currently prolonged until an appropriate intervention is provided.^8,9^ Strategies for early detection include active strategies, such as workshops for referral sources, which include healthcare (ie, community mental health or general healthcare services), educational, or community/governmental organization professionals.^10^ Additionally, general public awareness campaigns, including TV or radio appearances, theater advertisements, high school art contests, and sports sponsorships, are also potential outreach strategies to support early detection. Meanwhile, early *intervention* focuses on the provision of optimal treatments in these early phases of the psychotic disorder and is based on multidisciplinary teams of mental health professionals for individuals with early-onset psychosis, providing multimodal psychosocial and psychopharmacological interventions.

Duration of untreated psychosis (DUP) is usually defined as the period between the onset of psychosis and the start of treatment,^11^ although other definitions have been considered.^12,13^ DUP has been studied as a prognostic factor in schizophrenia. DUP has been associated with poor outcomes, including poor functioning.^8,14–18^ There is also highly suggestive evidence for a relationship between longer DUP and more severe positive symptoms, more severe negative symptoms, and lower chances of remission.^16^ Furthermore, there is suggestive evidence for an association between longer DUP and more severe global psychopathology.^16^ It has also been suggested that the association between DUP and psychosocial function may be an artifact of early detection, creating the illusion that early intervention is associated with improved outcomes.^19^ Hence, early detection programs may ascertain individuals with shorter DUP, less severe symptoms, and more individuals with affective psychosis.^20^

Interventions to reduce DUP based on early *detection* and early *intervention* in FEP have been developed^4,21^ based on the hypothesis that prolonged DUP leads to significant neurological and psychosocial damage that worsens the illness course of psychotic disorders.^22^ Early Intervention services (EIS) have been implemented to reduce DUP with promising results. In EIS, multidisciplinary teams of mental health professionals provide multimodal treatment, including different psychosocial and psychopharmacological interventions that are tailored to the needs of each patient.^4^ EIS is often considered the gold standard for the treatment of patients with early-phase psychosis.^4^

A meta-analysis published in this journal, including 16 studies up to April 2017, evaluated the efficacy of interventions to reduce DUP, with non-significant modest results (Hedges’ *g* = 0.12, *P* > .05).^14^ The frequency distributions of DUP are usually skewed, with outliers with very long DUP.^23^ Efforts to alter DUP by establishing early *detection* and intervention services have the potential to both detect individuals with FEP earlier and also to detect and intervene in those individuals that would have otherwise remained untreated.^24^ Thus, the inclusion of these patients could offer an unrealistically pessimistic picture of the impact of early *detection* efforts based on the alteration of DUP, artificially increasing DUP. Thus, other outcomes and correlates targeted by early *detection* and early *intervention* strategies need to be evaluated besides the reduction of DUP to understand the real-world impact of early *detection* and EIS in FEP. To our knowledge, this is the first systematic review and meta-analysis to evaluate the impact of early detection and intervention strategies on the reduction of DUP and mental health outcomes in first-episode psychosis. This study aimed to systematically review the evidence and provide meta-analytic data for (a) differences in DUP in individuals in early *detection* and *intervention* services vs individuals from the control group, (b) the efficacy of early *detection* strategies regarding real-world correlates at baseline (service entry), and (c) the efficacy of early *intervention* strategies on real-world outcomes at follow-up.

## METHODS

This systematic review was conducted according to the PRISMA 2020, (Supplementary table 1)^25^ and the MOOSE checklists (Supplementary table 2),^26^ following the EQUATOR Reporting Guidelines.^27^

### Search Strategy and Selection Criteria

A systematic search was used to identify relevant articles, and three qualified psychiatrists (GSP, AA, CAy) independently implemented a two-step literature search, looking at the titles and abstracts first, and the full text of the articles in a second step. The following terms were applied: (“first episode psych*” OR “FEP” OR “early-onset psychosis” OR “DUP” OR “duration untreated psych*”) AND (“reduc*” OR “decreas*” OR “early” OR “early intervention” OR “early detection” OR “service”). Researchers conducted the electronic search in PubMed and Web of Science database, incorporating the Web of Science Collection, BIOSIS Citation Index, KCI-Korean Journal, MEDLINE, Russian Science Citation Index, SciELO Citation Index, and Ovid/Psych databases from inception until February 01, 2023, without language restrictions. Second, we manually reviewed all references from the selected articles and extracted relevant additional articles. Articles identified were screened as abstracts, and after the exclusion of those which did not meet our inclusion criteria, the full texts of the remaining articles were assessed for eligibility, and decisions were made regarding their inclusion in the review.

The following inclusion criteria were used to select the articles: (a) individual studies, including conference proceedings; (b) conducted in individuals with FEP; (c) with both an intervention and a control group (including no intervention or historic control or alternative later intervention/treatment as usual—TAU—); (d) evaluating DUP in both groups as an outcome measure or a mediator (as mean ± *SD* or median) (definitions in Supplementary table 3); (e) reporting the impact of early *detection* or *intervention* in ≥1 relevant outcome for both groups; and (f) published in any language. Exclusion criteria were: (a) reviews, clinical cases, and protocols; (b) studies not reporting DUP in both groups; (c) studies without an independent control group; and (d) studies not reporting any outcome of interest. For the meta-analysis, additional inclusion criteria were: (a) full reporting of the correlates or outcomes of interest (ie, mean ± *SD* or %, see below) in both groups and (b) non-overlapping samples as defined by the study program and recruitment period.

### Outcome Measures and Data Extraction

Three qualified psychiatrists (AA, EMB, JSV), independently carried out data extraction, which was cross-checked by another author (GSP). The variables extracted included: author, year, program, country, sample size, mean age, % males, DUP, % affective psychosis, control characteristics, main correlates/outcomes (positive symptoms, negative symptoms, total psychopathology, depressive symptoms, quality of life, functioning, remission, relapse, employment, hospitalization) at baseline and longitudinally at the end of the study, quality assessment (see below), and key findings including other outcomes. DUP, positive symptoms, negative symptoms, total psychopathology, depressive symptoms, quality of life, and functioning were evaluated using continuous data (mean ± *SD*) in both groups. For the intervention strategies section, the results from baseline to the end of the study were evaluated. Remission, relapse, employment, and admissions rates were evaluated categorically (%) in both groups, at baseline and follow-up, respectively.

### Strategy for Data Synthesis

For the systematic review, we provided a narrative synthesis of the findings, structured around core outcomes and themes, excluding findings estimated meta-analytically, which were not repeated or expanded in this section. For the meta-analyses, the outcome measure was estimated when ≥3 studies were available by calculating the Hedges’ *g* for all correlates/outcomes to favor comparability. Notably, the meta-analysis of DUP and the meta-analytic correlates of early detection strategies are cross-sectional, while the analyses of meta-analytic outcomes of early intervention strategies are longitudinal and consider changes from baseline to follow-up, thus allowing the evaluation of changes on different scales for the same outcomes. Since high heterogeneity was expected, random effects meta-analyses were conducted.^28^ The presence of publication bias was assessed by Egger’s test,^29^ complemented by the “trim and fill” method to correct for the presence of missing studies when a risk of publication bias (ie, small sample bias) was detected. Heterogeneity among study point estimates was assessed using *Q* statistics. The proportion of the total variability in the effect size estimates was evaluated with the *I*^2^ index^30^ and considered statistically significant when *P* < .05. *I*^2^ > 50% is typically considered an indication of high variability in the effect size estimates. We conducted sub-analyses and meta-regression analyses for our three main research questions whenever ≥4 studies were available, including ≥2 studies per category in the categorical correlates/outcomes, to estimate the association between the efficacy of the intervention on each of the correlates/outcomes and (1) program continent (Europe vs America vs Australasia), (2) FEP diagnosis (% affective psychosis), (3) control content (TAU vs no intervention vs historic control), (4) mean age, (5) sex (% males), (6) DUP, (7) duration of the intervention—only for the intervention outcomes—, and (8) study quality (weak vs moderate vs strong). Further harmonization was not required for any of the outcomes as they were not dependent on different scales. We carried out “leave one out” analyses for the meta-analysis on differences in DUP in individuals in early detection and intervention services vs individuals from the control group. All *P* values reported in the meta-analyses were two-sided, with alpha = .05. Comprehensive Meta-analysis (CMA) V3^31^ was used to perform the analyses.

### Risk of Bias (Quality) Assessment

The study quality was assessed using the “Effective Public Health Practice Project” (EPHPP),^32,33^ as most studies were expected not to be randomized. The following items were evaluated as good, fair, or poor: (a) selection bias, (b) design, (c) confounders, (d) blinding, (e) data collection, and (f) dropouts. The overall quality was rated in three categories: weak, moderate, or strong. Studies were evaluated as strong when none of the items was rated as poor; moderate if one item was rated as poor; and weak if ≥2 a–f items were evaluated as poor. After discussion with the corresponding author, 100% discrepancies were resolved.

## Results

**Fig. 1.**
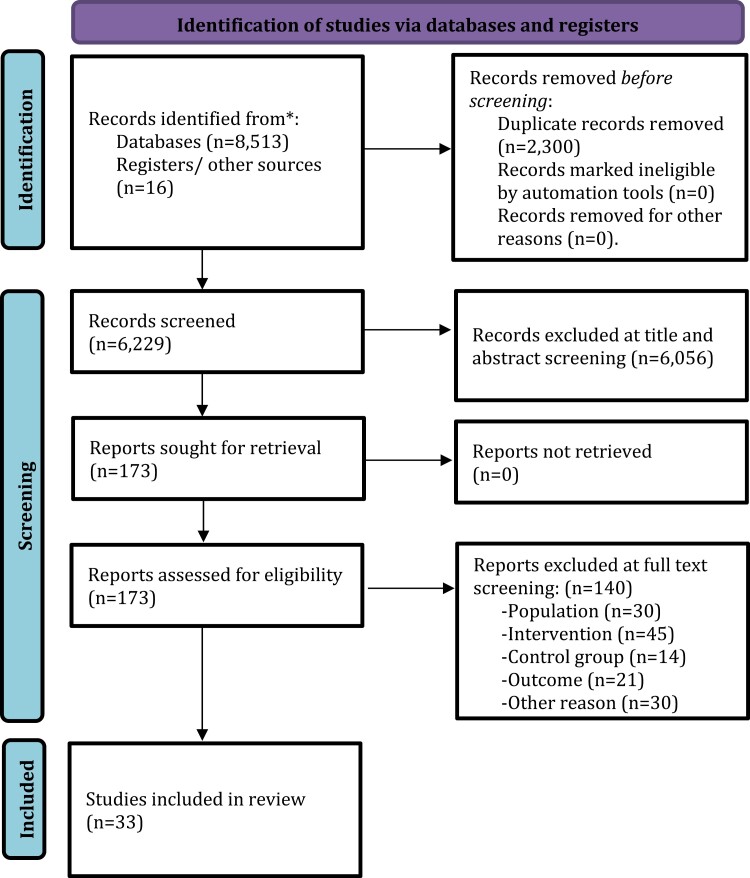
Preferred Reporting Items for Systematic Reviews and Meta-analyses (PRISMA) flowchart outlining study selection process.

The literature search yielded 6229 citations, which were screened for eligibility, and 33 articles were finally included in the systematic review and meta-analysis (figure 1). The database included 9093 individuals: 5288 in the intervention group and 3805 in the control group. The total sample size (including both intervention and control groups) of the included studies ranged from 65^34^ to 1234^35^ individuals (Supplementary table 4). The mean age of the sample ranged from 21.2^35,36^ to 31.1^37^ years. The proportion of males ranged from 45.3%^38^ to 81.5%.^39^

### Meta-analysis of DUP

**Fig. 2.**
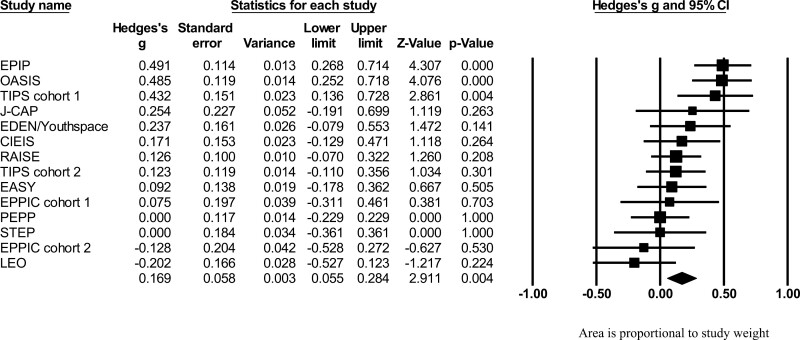
Forest plot of strategies to reduce DUP. Area is proportional to study weight.

Altogether, 14 cohorts from 12 different EIS (*n* = 2938) provided meta-analytic data to compare DUP in an intervention (*n* = 1616) vs a control group (*n* = 1312). We found that the early detection/intervention group reduced DUP (*g* = 0.168, 95% CI = 0.055–0.283) compared to the control group, with a small effect size (figure 2). Heterogeneity was significant among the services (*Q* = 29.109 *P* = .006 *I* = 55.34%). Publication bias was not detected (Egger’s test = 1.83, *P* = .309). In “leave one out” analyses, the statistical significance did not change in any scenario: the maximum ES was when LEO was removed (*g* = 0.197, 95% CI = 0.087–0.306), and the minimum ES was when OASIS was removed (*g* = 0.142, 95% CI = 0.033–0.267).

### Meta-analytic Results of Early Detection Strategies

**Fig. 3.**
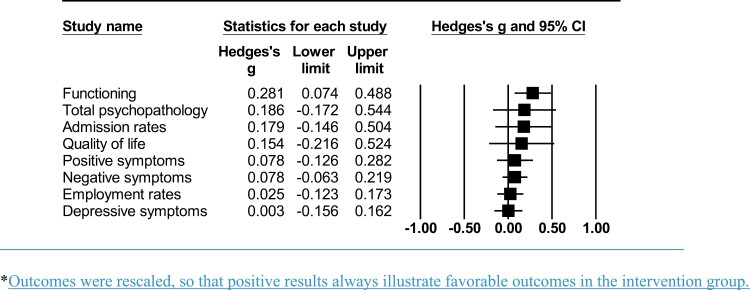
Meta-analytic outcomes of early detection strategies. *Outcomes were rescaled, so that positive results always illustrate favorable outcomes in the intervention group.

Studies reported (in descending order of frequency) on negative symptoms (*k* = 10, *n* = 2255), positive symptoms (*k* = 8, *n* = 1637), functioning (*k* = 8, *n* = 2192), total psychopathology (*k* = 7, *n* = 1934), employment rates (*k* = 7, *n* = 2554), quality of life (*k* = 4, *n* = 1002), depressive symptoms (*k* = 3, *n* = 610), and admission rates (*k* = 3, *n* = 754) (table 1, figure 3).

**Table 1.** Meta-analytic Outcomes of Early Detection Strategies

Outcome	*k* Studies	*n* INT	*n* CTRL	Hedges’ *g*			*Z* Scores	*P*	Test for Heterogeneity			Egger’s Test	
				Mean	95% CI				*Q*	*I* ^2^	*P*	*T* Values	*P*
Functioning^a^	8 (10)	1182	1010	0.281	0.073	0.488	2.653	.008	27.310	74.368	<.001	0.209	.841
Total psychopathology^b^	7 (10)	1032	902	0.186	−0.173	0.546	1.016	.310	49.654	87.916	<.001	0.307	.771
Admission rates	3 (3)	348	406	0.179	−0.146	0.504	1.08	.280	5.747	65.202	.056	0.143	.908
Quality of life	4 (5)	546	456	0.154	−0.217	0.525	0.812	.417	13.193	77.261	.004	4.182	.053
Positive symptoms^c^	8 (14)	809	828	0.078	−0.126	0.283	0.749	.454	26.951	74.027	<.001	0.367	.726
Negative symptoms^d^	10 (16)	1231	1024	0.078	−0.064	0.219	1.078	.281	20.719	56.559	.014	0.638	.541
Employment rates	7 (7)	1307	1247	0.025	−0.124	0.173	0.324	.746	7.585	20.901	.270	0.262	.804
Depressive symptoms^e^	3 (3)	328	282	0.003	−0.157	0.162	0.031	.975	0.059	0.000	.971	0.333	.795

*Note*: ^a^Functioning was evaluated with the Global Assessment of Functioning (GAF),^9^ the Social and Occupational Functioning Assessment Scale (SOFAS)^10^ or the Global Functioning: Role (GFR); Global Functioning: Social (GFS).^11,12^

^b^Total psychopathology was evaluated with the Positive and Negative Syndrome Scale (PANSS)^2^ or the Brief Psychiatric Rating Scale (BPRS).^4^

^c^Positive symptoms were evaluated with the Positive and Negative Syndrome Scale (PANSS),^2^ the Scale for the Assessment of Positive Symptoms (SAPS)^3^ or the Brief Psychiatric Rating Scale (BPRS).^4^

^d^Negative symptoms were evaluated with the Positive and Negative Syndrome Scale (PANSS)^2^ or the Scale for the Assessment of Negative Symptoms (SANS).^5^

^e^Depressive symptoms were evaluated with the Hamilton Rating Scale for Depression (HAM-D),^6^ the Calgary Depression Scale for Schizophrenia (CDSS)^7^ or the Beck Depression Inventory (BDI).^8^ Bold values indicate *P* < 0.05.

Compared to individuals in the control group, individuals in the early *detection* group had better functioning levels (*g* = 0.281, 95% CI = 0.073–0.488) at baseline. Total psychopathology (*g* = 0.186, 95% CI = −0.173 to 0.546), admission rates (*g* = 0.179, 95% CI = −0.146 to 0.504), quality of life (*g* = 0.154, 95% CI = −0.217 to 0.525), positive symptoms (*g* = 0.078, 95% CI = −0.126 to 0.283), negative symptoms (*g* = 0.078, 95% CI = −0.064 to 0.219), employment rates (*g* = 0.025, 95% CI = −0.124 to 0.173), and depressive symptoms (*g* = 0.003, 95% CI = −0.157 to 0.162), did not differ between both groups (table 1, figure 3) (forest plots available in Supplementary figure 1).

### Meta-analytic Outcomes of Early Intervention Strategies

**Fig. 4.**
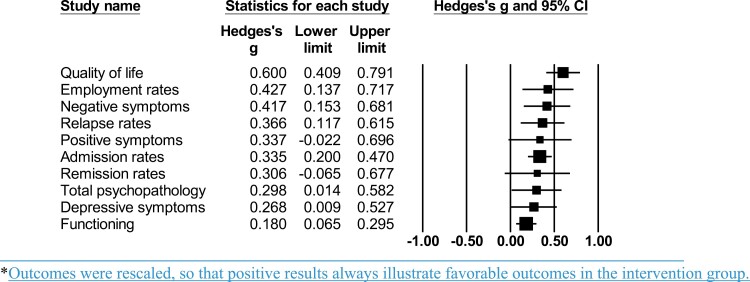
Meta-analytic outcomes of early intervention strategies. *Outcomes were rescaled, so that positive results always illustrate favorable outcomes in the intervention group.

Studies reported (in descending order of frequency) on negative symptoms (*k* = 8, *n* = 1499), positive symptoms (*k* = 7, *n* = 1490), total psychopathology (*k* = 7, *n* = 1327), functioning (*k* = 6, *n* = 1452), admission rates (*k* = 5, *n* = 490), quality of life (*k* = 4, *n* = 1061), remission rates (*k* = 4, *n* = 821), depressive symptoms (*k* = 3, *n* = 393), relapse rates (*k* = 3, *n* = 380), and employment rates (*k* = 3, *n* = 259) (table 1, figure 4). Compared to the control group, early *intervention* improved outcomes longitudinally including quality of life (*g* = 0.600, 95% CI = 0.408–0.791), increased employment rates (*g* = 0.423, 95% CI = 0.134–0.712), improved negative symptoms (*g* = 0.417, 95% CI = 0.153–0.682), decreased relapse rates (*g* = 0.364, 95% CI = 0.117–0.612), reduced hospitalizations (*g* = 0.335, 95% CI = 0.198–0.468), improved total psychopathology (*g* = 0.298, 95% CI = 0.014–0.582), improved depressive symptoms (*g* = 0.268, 95% CI = 0.008–0.528), and improved functioning (*g* = 0.180, 95% CI = 0.065–0.295) at follow-up. No group differences were found for positive symptoms (*g* = 0.337, 95% CI = −0.022 to 0.696) and remission rates (*g* = 0.306, 95% CI = −0.066 to 0.677 corrected to *g* = 0.180, 95% CI = −0.193, 0.552) (table 2, figure 4) (forest plots available in Supplementary figure 2).

**Table 2.** Meta-analytic Outcomes of Early Intervention Strategies

Outcome	*k* Studies	*n* INT	*n* CTRL	Hedges’ *g*			*Z* Score	*P*	Test for Heterogeneity			Egger’s Test	
				Mean	95% CI				*Q*	*I* ^2^	*P*	*T* Values	*P* Values
Quality of life^a^	4 (5)	575	486	0.600	0.408	0.791	6.146	**<.001**	3.737	19.726	.291	1.890	.199
Employment rates	3 (3)	132	127	0.427	0.135	0.718	2.869	**.004**	0.376	0.000	.829	0.096	.939
Negative symptoms^b^	8 (13)	849	650	0.417	0.153	0.682	3.091	**.002**	41.017	82.934	<.001	0.374	.721
Relapse rates	3 (3)	194	186	0.366	0.117	0.616	2.882	**.004**	0.223	0.000	.894	0.295	.817
Positive symptoms^c^	7 (12)	813	677	0.337	−0.022	0.696	1.841	.066	63.406	90.537	<.001	0.788	.466
Admission rates	5 (5)	246	244	0.335	0.198	0.468	4.057	**<.001**	4.408	9.248	.354	3.617	.036^d^
Remission rates	4 (4)	426	395	0.306	−0.066	0.677	1.613	.107	9.772	69.300	.021	18.656	.003^e^
Total psychopathology^f^	7 (10)	677	650	0.298	0.014	0.582	2.054	**.040**	27.990	78.564	<.001	0.080	.939
Depressive symptoms^g^	3 (3)	196	197	0.268	0.008	0.528	2.019	**.043**	3.029	33.968	.220	3.994	.156
Functioning^h^	6 (7)	803	649	0.180	0.065	0.295	3.062	**.002**	2.155	0.000	.827	1.14	.312

*Note*: ^a^Quality of Life was evaluated with the Quality of Life Scale (QLS),^13^ the Short Form Health Survey (SF-12)^14^ or the World Health Organization Quality of Life (WHO-QoL).^15^

^b^Negative symptoms were evaluated with the Positive and Negative Syndrome Scale (PANSS)^2^ or the Scale for the Assessment of Negative Symptoms (SANS).^5^

^c^Positive symptoms were evaluated with the Positive and Negative Syndrome Scale (PANSS),^2^ the Scale for the Assessment of Positive Symptoms (SAPS)^3^ or the Brief Psychiatric Rating Scale (BPRS).^4^

^d^Funnel plot inspection revealed asymmetry to the right. Due to the lack of small sample bias, we did not adjust our results with the trim-and-fill method.

^e^Funnel plot inspection revealed asymmetry to the left. Small sample bias was corrected with the trim-and-fill method: to *g* = 0.180, 95% CI = −0.193 to 0.552.

^f^Total psychopathology was evaluated with the Positive and Negative Syndrome Scale (PANSS)^2^ or the Brief Psychiatric Rating Scale (BPRS).^4^

^g^Depressive symptoms were evaluated with the Hamilton Rating Scale for Depression (HAM-D),^6^ the Calgary Depression Scale for Schizophrenia (CDSS)^7^ or the Beck Depression Inventory (BDI).^8^

^h^Functioning was evaluated with the Global Assessment of Functioning (GAF),^9^ the Social and Occupational Functioning Assessment Scale (SOFAS)^10^ or the Global Functioning: Role (GFR); Global Functioning: Social (GFS).^11,12^ Bold values indicate *P* < 0.05.

### Other Non-meta-analytic Outcomes of Early Detection and Intervention Strategies

After implementing early *detection* strategies, differences were found in the referral patterns,^20,40^ although not consistently.^41^ Police referrals decreased by 15.2% (χ^2^ = 10.5, *P* = .001),^40^ while self and family referrals increased by 10.7% (χ^2^ = 3.5, *P* = .04)^40^ in the early *detection* group. Individuals with FEP in the early *detection* group were more likely to get clinical care without previous mental health services contact (*P* = .003).^6^ Furthermore, early *detection* services had relatively more patients with affective psychosis (χ^2^ = 4.011, *P* = .028),^20^ and low socioeconomic status (χ^2^ = 8.659, *P* = .003),^20^ whereas premorbid functioning did not differ between the early *detection* and the control group.^42^

Regarding early *intervention* strategies, some studies did not find significant group differences in help-seeking attempts,^43^ while others found advantages for the intervention vs the control group regarding decreased delay in help-seeking (*P* = .01)^44^ and in reaching mental health services (*P* = .003).^44^ Moreover, compared to the control group, individuals with FEP in the early *intervention* group had more friends after 1 year of care (*P* = .02),^45^ greater improvements in cognitive symptoms (*P* < .001),^46^ and perceived autonomy (*P* < .01)^47^ after 2 years, and were less likely to live in supported housing after 5 years (*P* = .02).^48^ Compared to the control group, individuals with FEP in the intervention group had lower admission rates and days hospitalized^48,49^ (although not consistently^50^), and were less frequently admitted under the Mental Health Act^50^ or in locked units^51^ (all *P* < .05). However, no intervention vs control group differences were found in the rates of police involvement and use of seclusion.^51^ Individuals in the early *intervention* vs control group had fewer suicide attempts^49^ and death by suicide^35,49,52^ (all *P* < .05), lower rates of antipsychotics^40,53^ (particularly first-generation antipsychotics^37^) and at lower dose,^40^ with lower maximum initial dosages,^54^ as well as lower rates of benzodiazepines^40^ and anticholinergic medications.^40^ Satisfaction with care was high in the intervention group (3.9/5 for patients and 4/5 for relatives).^53^ However, family satisfaction, after adjusting for baseline characteristics, was not higher anymore in the intervention vs the control group in one of the included studies.^55^ In the early *intervention* vs control group, adherence to comprehensive community care was higher,^56^ dropout rates lower,^55^ and mental health service costs were lower 8 years after the early *intervention* ended (*P* = .01).^34^ A summary of the potential additional benefits detected in our systematic review can be found in Supplementary figure 5.

### Heterogeneity, Publication Bias, and Meta-regression Analyses

Heterogeneity across the included studies was statistically significant in 5/8 correlates in the early *detection* group, ranging from 56.6% to 87.9% in those correlates. Meanwhile, heterogeneity was statistically significant in 4/10 outcomes in the intervention group, ranging from 69.3% to 90.5% in those outcomes. Publication bias was not detected in any of the correlates at the time of service contact in the early *detection* strategies. Heterogeneity was detected in two of the early *intervention* strategy outcomes, ie, admissions rates (*P* = .036) and remission rates (*P* = .003).

Regarding admission rates, funnel plot inspection revealed asymmetry to the right. Due to the lack of small sample bias, we did not adjust results with the trim-and-fill method, and the original value was maintained. Regarding remission rates, funnel plot inspection revealed asymmetry to the left. Small effect bias was thus corrected with the trim-and-fill method, decreasing the effect size from *g* = 0.306 (CI = −0.066 to 0.677) to *g* = 0.180 (95% CI = −0.193 to 0.552) (funnel plots available in Supplementary figures 3 and 4).

In meta-regression analyses of DUP, none of the variables evaluated was statistically significant (all *P* > .05). In meta-regression analyses of early *detection* correlates, greater efficacy of early *detection* strategies for the total psychopathology outcome was associated with a higher mean age (β = .124, *P* = .020), and a lower % of males (β = −.035, *P* = .024). Greater efficacy of the interventions for quality of life was associated with a higher proportion of individuals with affective psychosis (β = 5.599, *P* = .011), while greater efficacy for functioning was associated with a higher mean age (β = 0.061, *P* = .029). There was no significant association between other evaluated moderating factors including DUP, continent, control content, and quality of the studies with other early *detection* correlates (all *P* > .05) (Supplementary table 5). For early *intervention* outcomes, a stronger decrease in the DUP was associated with a greater improvement in the intervention vs control group in quality of life (β = .025, *P* = .023) but not the severity of positive symptoms (β = −.067, *P* = .431), negative symptoms (β = .053, *P* = .151), overall psychopathology (β = .0044, *P* = .802), functioning (β = .005, *P* = .530), remission (β = .040, *P* = .178), or number of subsequent admissions (β = −.014, *P* = .234). A higher % of males (β = .080, *P* = .014) was associated with a greater improvement in remission rates. There was no significant association between other evaluated moderating factors with other early *intervention* outcomes including % affective psychosis, control content, age, or quality of the study (all *P* > .05) (Supplementary table 6).

### Quality Assessment

The quality of the included studies ranged from weak (*k* = 16, 48.5%) to strong (*k* = 3, 9.1%). The item most frequently reported as good was data collection (*k* = 29, 87.9%); The item most frequently reported as poor was blinding (*k* = 29, 87.9%) (Supplementary figure 6).

## Discussion

To the best of our knowledge, this is the first systematic review and meta-analysis to comprehensively evaluate the role of DUP as a treatment target and moderator of early *detection* and *intervention* strategies for first-episode psychosis. We aimed to look at the impact of early *detection* and *intervention* strategies on both DUP and related real-world outcomes. We described the results from 33 studies narratively and performed different meta-analyses with some of the most clinically relevant and most reported outcomes. We found that the intervention group reduced DUP (*g* = 0.168) compared to the control group. While from the evaluated variables, the early *detection* group only had better functioning levels (*g* = 0.281) at service engagement/baseline than the control group, the early *intervention* group was able to improve 8/10 outcomes: quality of life (*g* = 0.600), employment rates (*g* = 0.423), negative symptoms (*g* = 0.417), relapse rates (*g* = 0.364), admission rates (*g* = 0.335), total psychopathology (*g* = 0.298), depressive symptoms (*g* = 0.268), and functioning levels (*g* = 0.180) compared to the control group.

We evaluated the role of DUP as a determinant of mental health for individuals with FEP. We found that the early detection/intervention group reduced DUP compared to the control group, with a small effect size. Our updated results are somewhat more promising than those from a previous meta-analysis reporting changes in DUP,^14^ which found similar effect sizes (*g* = 0.12), but did not detect significant differences between the groups (*P* > .05). However, these two meta-analyses both suggest that the current impact of early *detection* strategies on DUP is limited. We believe that there are some individuals with very long DUP,^23^ that can only reach care with intensive efforts from professionals, which may be a limiting factor that prevents early *detection* strategies from having a greater impact on DUP. In fact, one of the included studies found that while only 3.4% of the individuals in the control group had very long (>3 years) DUP, this number reached 15.0% in the intervention group (*P* = .005).^24^ However, we cannot rule out that some of the strategies may have simply failed in their attempt to reduce DUP in individuals with FEP. In any case, evaluating the impact of the efforts to reduce DUP on mental health outcomes in first-episode psychosis through early detection and intervention strategies is an important indication of their real-world effectiveness. Our results support the implementation of EIS aiming to shorten DUP with both an early detection and intervention component,^57^ even if the impact on DUP seems limited. It is also possible that robust, comprehensive treatments in FEP improve outcomes regardless of DUP changes. Our superior results of *early intervention* strategies (improving 8/10 outcomes) compared to *early detection* strategies would support this hypothesis.

Early *detection* strategies resulted in better functioning levels at baseline compared to individuals in the control group. However, the groups did not differ regarding total psychopathology, admission rates, quality of life, positive symptoms, negative symptoms, employment rates, and depressive symptoms. One hypothesis would be that early detection may result in individuals entering services prior to more severe functional deterioration. However, although functioning is critical in psychosis and schizophrenia,^58^ it seems that current *detection* strategies fail to detect individuals with FEP before more relevant symptoms and other poor outcomes develop. As discussed above, it is possible that the *detection* of more severely affected individuals that otherwise would have remained without treatment may have played a significant role. However, it is also possible and desirable to refine actual *detection* strategies. For instance, it seems that information campaigns,^59^ especially if they are multi-focus^60^ in nature, can optimize *detection* strategies. Other strategies, like targeted health education to reduce DUP by helping to better identify signs of mental illness, have also shown promising results,^61^ since ongoing training correlated with a DUP reduction.^61^ Barriers to early *detection* include difficulties in detecting signs of early psychosis,^6^ worries about stigma or coercive treatment,^6^ and family difficulties in judging the disease appropriately.^62^ Moreover, developing local networking activities targeting professionals in the education and primary healthcare sectors may help improve pathways to care.^63^ A longer DUP has been associated with family members blaming puberty or ideology for the psychosis rather than considering a mental health problem.^62^ This highlights the importance of outreach strategies and information campaigns in the community. Regarding the best *detection* strategies to reduce DUP and improve *detection* correlates, EIS typically provides treatment and support for both individuals experiencing psychosis and individuals who are at high risk of developing psychosis.^64^ Establishing standalone services for Clinical High Risk for Psychosis (CHR-P) with both an early detection and early detection component seems to be the most effective method for reducing DUP,^14^ although the amount of available evidence is limited. *Detection*^65^ of individuals at CHR-P and early interventions^66^ directed towards the prevention of psychosis,^67^ have the potential to maximize the benefits of early *interventions* in psychosis,^3,4^ favoring an earlier *detection* and potentially a reduction in the DUP.

In our meta-analysis, compared to the control group, early *interventions* improved most clinical outcomes. Previous evidence suggests that EIS, even when these do not have a specific early *detection* component, can reduce DUP.^68^ Our results align with a previous meta-analysis that found that EIS was superior to treatment as usual regarding each of the 15 meta-analysed outcomes.^4^ Although we did not limit the included studies to randomized interventions,^4^ apart from to those reporting DUP, our effect sizes were similar (small to medium). This finding suggests that the provision of early psychosocial and psychopharmacological interventions is clearly beneficial for individuals with FEP, possibly regardless of DUP. Interestingly, although previous evidence suggests that a delayed start of antipsychotic medication could lead to an increased manifestation and severity of positive symptoms in the long term,^69^ the early *intervention* did not have a significant impact on positive symptoms, according to our results. We found that rates and doses of antipsychotics may be lower in the early *intervention* group,^40,53,54^ probably in an attempt to minimize side effects.^70–72^ The effect of this lower antipsychotic rate remains unknown, but recently several meta-analyses have shown that lower than therapeutic antipsychotic doses or dose reduction during maintenance treatment are associated with a higher risk of relapse and hospitalization.^73–77^ In contrast, the number of studies evaluating remission rates was low (*k* = 4), limiting our power for this analysis, and the confidence intervals for the remission rates also crossed the null hypothesis line.

In the systematic review, other potential benefits of early *detection* and early *intervention* strategies for other outcomes are suggested, although due to limited data, this was not accompanied by meta-analytical evidence. Among these outcomes, a decrease in potentially traumatic experiences, such as police referrals,^40^ admissions in locked units,^51^ or admissions under the Mental Health Act,^50^ could be beneficial, as childhood and adult adversities have shown to be associated with increased psychotic symptoms in individuals with psychotic disorders,^78^ and increased risk of developing psychosis.^79,80^ Among the evaluated outcomes, the benefits of EIS on suicide rates^35,49,52^ and on service users’ satisfaction,^53^ pivotal to favor engagement and decrease dropout rates, are notable. Finally, from a management, resource allocation, and funding perspective,^4^ it is relevant that the costs of EIS seem to be lower than the control group costs,^34^ particularly due to lower inpatient costs.^81^

According to our results, early *detection* strategies were more effective in older female individuals for total psychopathology, in individuals with affective psychosis for quality of life, and in older individuals for functioning. Meanwhile, early *intervention* strategies were more effective in individuals with a more pronounced decrease in DUP for quality of life and in older individuals for remission rates. These findings suggest that some interventions may improve some particular outcomes more easily in individuals with certain characteristics, while in others, achieving this benefit may be more challenging. Precision or personalized medicine considers individual variability when establishing, targeting, and delivering an intervention.^82,83^ Therefore, the need to stratify interventions according to individual characteristics has been suggested to improve outcomes.^84,85^ In fact, in early *intervention* for psychosis, individual characteristics may help detect patient subgroups requiring an adaptation in the duration of the interventions or in its specific content or may suggest the need for higher-intensity interventions.^4^ The implementation of EIS varies significantly worldwide. For instance, there is almost complete nationwide EIS coverage in Denmark and England, while almost no services are available in many other European countries and low-income countries. It has been suggested that these differences are likely due to local traditions rather than science.^57^

The current study has several limitations. First, the number of available studies was limited, especially for depressive symptoms and admission rates in the early *detection* correlates, and for depressive symptoms, relapse rates, and employment rates for early *intervention* outcomes. Other outcomes (eg, police involvement) were not meta-analysed due to lack of data but included in the systematic review. However, the database was extensive and sufficiently powered to evaluate a broad range of correlates/outcomes. Second, some of the studies had a suboptimal design, including the use of historical control groups due to ethical and implementation reasons. Consequently, 48.5% of the studies had a weak study quality, according to the EPHPP. Particularly, for 87.9% of the included studies, there was no blinding, or this feature was not reported. We conducted meta-regression analyses for both the quality of the studies and the control content and did not find any association between these factors and evaluated correlates/outcomes. Third, we only meta-analysed studies in which DUP for both groups was provided as mean ± *SD*, as we were not able to pool median DUP following expert statistical advice. Studies using median DUP were included for meta-analytic results of early detection strategies and meta-analytic outcomes of early intervention strategies. However, this approach has allowed us to obtain more homogeneous and comparable measures. Fourth, heterogeneity was significant for DUP and other outcomes, as detailed in the manuscript. Different factors may have influenced the observed heterogeneity, including the setting where the intervention was conducted, and the duration of the intervention. Nevertheless, heterogeneity is common in real-world scenarios, possibly being reflective of our having captured an authentic picture. Fifth, we could not determine for how long it would be appropriate for the interventions to be provided or their differential efficacy for discrete time periods. However, the duration of the intervention did not have a significant impact on any of the outcomes according to the meta-regression analyses. Sixth, we evaluated nineteen outcomes, but we did not apply the multiple-testing correction. Note, as per the Cochrane Handbook, that one in 20 independent statistical tests will be statistically significant at a 5% significance level.^86^ Seventh, due to heterogeneity and the limited number of included studies, we could not report on the outcomes of specific detection or intervention strategies. Furthermore, all the studies evaluation early intervention outcomes contain early detection components aiming to reduce DUP. Finally, the thresholds regarding DUP varied, and we could not establish the target or minimum reduction of DUP, which would have a specific or threshold impact on mental health outcomes. The definitions of DUP were also different. Notably, defining and reporting DUP presents reliability challenges due to the presence of different levels of insight in patients, blurry borders between attenuated and full psychosis symptoms, and different levels of acuity and severity during the onset of symptoms. However, a meta-analysis of 369 studies found no differences in DUP values according to the definition.^87^ We conducted additional meta-regression analyses to evaluate any association between the analysed outcomes and various factors, including the continent where the intervention was carried out, % of study participants with affective psychosis, control content, mean participant age, % of males, DUP, and duration of the intervention.

## Conclusion

When comparing strategies targeting DUP and control groups, the impact of early *detection* strategies on DUP and other outcomes is limited. However, the impact of early *intervention* on the outcomes evaluated, including quality of life, employment, and relapse rates, is significant. Our results support the implementation of EIS with both an early detection and intervention component using robust and comprehensive treatments, even if the impact on DUP is limited. Further research into specific early detection and intervention components using culturally sensitive approaches is required.

## Supplementary Material

Supplementary material is available at https://academic.oup.com/schizophreniabulletin/.

sbae017_suppl_Supplementary_Material

## References

[1] Vos T , Flaxman AD, Naghavi M, et al. Years lived with disability (YLDs) for 1160 sequelae of 289 diseases and injuries 1990–2010: a systematic analysis for the global burden of disease study 2010. Lancet. 2012;380(9859):2163–2196. 23245607 10.1016/S0140-6736(12)61729-2 PMC6350784

[2] Tandon R , Nasrallah HA, Keshavan MS. Schizophrenia, “just the facts” 4. Clinical features and conceptualization. Schizophr Res. 2009;110(1–3):1–23. 19328655 10.1016/j.schres.2009.03.005

[3] Fusar-Poli P , McGorry PD, Kane JM. Improving outcomes of first-episode psychosis: an overview. World Psychiatry. 2017;16(3):251–265. 28941089 10.1002/wps.20446 PMC5608829

[4] Correll CU , Galling B, Pawar A, et al. Comparison of early intervention services vs treatment as usual for early-phase psychosis a systematic review, meta-analysis, and meta-regression. Jama Psychiatry. 2018;75(6):555–565. 29800949 10.1001/jamapsychiatry.2018.0623 PMC6137532

[5] Birchwood M , Todd P, Jackson C. Early intervention in psychosis—the critical period hypothesis. Br J Psychiatry. 1998;172:53–59. 9764127

[6] Lloyd-Evans B , Sweeney A, Hinton M, et al. Evaluation of a community awareness programme to reduce delays in referrals to early intervention services and enhance early detection of psychosis. Bmc Psychiatry. 2015;15:98. 25934413 10.1186/s12888-015-0485-y PMC4424506

[7] Lynch S , McFarlane WR, Joly B, et al. Early detection, intervention and prevention of psychosis program: community outreach and early identification at six US sites. Psychiatr Serv (Washington, D.C.). 2016;67(5):510–516. 10.1176/appi.ps.201300236 26766751

[8] Perkins DO , Gu H, Boteva K, Lieberman JA. Relationship between duration of untreated psychosis and outcome in first-episode schizophrenia: a critical review and meta-analysis. Am J Psychiatry. 2005;162(10):1785–1804. 16199825 10.1176/appi.ajp.162.10.1785

[9] Lloyd-Evans B , Crosby M, Stockton S, et al. Initiatives to shorten duration of untreated psychosis: systematic review. Br J Psychiatry. 2011;198(4):256–263. 21972275 10.1192/bjp.bp.109.075622

[10] Salazar de Pablo G , Estradé A, Cutroni M, Andlauer O, Fusar-Poli P. Establishing a clinical service to prevent psychosis: what, how and when? Systematic review. Transl Psychiatry. 2021;11(1):43. 33441556 10.1038/s41398-020-01165-x PMC7807021

[11] Hegelstad WT , Larsen TK, Auestad B, et al. Long-term follow-up of the TIPS early detection in psychosis study: effects on 10-year outcome. Am J Psychiatry. 2012;169(4):374–380. 22407080 10.1176/appi.ajp.2011.11030459

[12] Compton M , Carter T, Bergner E, et al. Defining, operationalizing and measuring the duration of untreated psychosis: advances, limitations and future directions. Early Interv Psychiatry. 2007;1:236–250.

[13] Golay P , Alameda L, Baumann P, et al. Duration of untreated psychosis: impact of the definition of treatment onset on its predictive value over three years of treatment. J Psychiatr Res. 2016;77:15–21. 26950643 10.1016/j.jpsychires.2016.02.017

[14] Oliver D , Davies C, Crossland G, et al. Can we reduce the duration of untreated psychosis? A systematic review and meta-analysis of controlled interventional studies. Schizophr Bull. 2018;44(6):1362–1372. 29373755 10.1093/schbul/sbx166 PMC6192469

[15] Penttila M , Jaaskelainen E, Hirvonen N, Isohanni M, Miettunen J. Duration of untreated psychosis as predictor of long-term outcome in schizophrenia: systematic review and meta-analysis. Br J Psychiatry. 2014;205(2):88–94. 25252316 10.1192/bjp.bp.113.127753

[16] Howes O , Whitehurst T, Shatalina E, et al. The clinical significance of duration of untreated psychosis: an umbrella review and random-effects meta-analysis. World Psychiatry. 2021;20:75–95. 33432766 10.1002/wps.20822 PMC7801839

[17] Díaz-Caneja CM , Pina-Camacho L, Rodríguez-Quiroga A, Fraguas D, Parellada M, Arango C. Predictors of outcome in early-onset psychosis: a systematic review. Npj Schizophr. 2015;1:14005. 27336027 10.1038/npjschz.2014.5 PMC4849440

[18] Fraguas D , Del Rey-Mejías A, Moreno C, et al. Duration of untreated psychosis predicts functional and clinical outcome in children and adolescents with first-episode psychosis: a 2-year longitudinal study. Schizophr Res. 2014;152(1):130–138. 24332406 10.1016/j.schres.2013.11.018

[19] Jonas KG , Fochtmann LJ, Perlman G, et al. Lead-time bias confounds association between duration of untreated psychosis and illness course in schizophrenia. Am J Psychiatry. 2020;177(4):327–334. 32046533 10.1176/appi.ajp.2019.19030324 PMC10754034

[20] Malla A , Jordan G, Joober R, et al. A controlled evaluation of a targeted early case detection intervention for reducing delay in treatment of first episode psychosis. Soc Psychiatry Psychiatr Epidemiol. 2014;49(11):1711–1718. 24902532 10.1007/s00127-014-0893-1

[21] Lieberman JA , Small SA, Girgis RR. Early detection and preventive intervention in schizophrenia: from fantasy to reality. Am J Psychiatry. 2019;176(10):794–810. 31569988 10.1176/appi.ajp.2019.19080865

[22] Salazar de Pablo G , Guinart D, Correll CU. What are the physical and mental health implications of duration of untreated psychosis? Eur Psychiatry. 2021;64(1):e46. 33775268 10.1192/j.eurpsy.2021.22 PMC8316447

[23] Johannessen JO , McGlashan TH, Larsen TK, et al. Early detection strategies for untreated first-episode psychosis. Schizophr Res. 2001;51(1):39–46. 11479064 10.1016/s0920-9964(01)00237-7

[24] Krstev H , Carbone S, Harrigan SM, Curry C, Elkins K, McGorry PD. Early intervention in first-episode psychosis—the impact of a community development campaign. Soc Psychiatry Psychiatr Epidemiol. 2004;39(9):711–719. 15672291 10.1007/s00127-004-0798-5

[25] Moher D , Liberati A, Tetzlaff J, Altman DG, Group P. Preferred reporting items for systematic reviews and meta-analyses: the PRISMA statement. BMJ. 2009;339:b2535. 19622551 10.1136/bmj.b2535 PMC2714657

[26] Stroup DF , Berlin JA, Morton SC, et al. Meta-analysis of observational studies in epidemiology: a proposal for reporting. Meta-analysis Of Observational Studies in Epidemiology (MOOSE) group. JAMA. 2000;283(15):2008–2012. 10789670 10.1001/jama.283.15.2008

[27] Altman DG , Simera I, Hoey J, Moher D, Schulz K. EQUATOR: reporting guidelines for health research. Lancet. 2008;371(9619):1149–1150. 18395566 10.1016/S0140-6736(08)60505-X

[28] DerSimonian R , Laird N. Meta-analysis in clinical trials. Control Clin Trials. 1986;7(3):177–188. 3802833 10.1016/0197-2456(86)90046-2

[29] Egger M , Davey Smith G, Schneider M, Minder C. Bias in meta-analysis detected by a simple, graphical test. BMJ (Clin Res Ed). 1997;315(7109):629–634. 10.1136/bmj.315.7109.629 PMC2127453 9310563

[30] Lipsey M , Wilson D. Practical Meta-analysis. Thousand Oaks, CA: Sage Publications; 2000.

[31] Borenstein M , Hedges L, Higgins J, Rothstein H. *Comprehensive Meta-Analysis Version 3* [computer program]. Version. Englewood, NJ: Biostat; 2013.

[32] Thomas BH , Ciliska D, Dobbins M, Micucci S. A process for systematically reviewing the literature: providing the research evidence for public health nursing interventions. *Worldviews Evid Based Nurs.* 2004;1(3):176–184. 17163895 10.1111/j.1524-475X.2004.04006.x

[33] Armijo-Olivo S , Stiles CR, Hagen NA, Biondo PD, Cummings GG. Assessment of study quality for systematic reviews: a comparison of the Cochrane Collaboration Risk of Bias Tool and the Effective Public Health Practice Project Quality Assessment Tool: methodological research. J Eval Clin Pract. 2012;18(1):12–18. 20698919 10.1111/j.1365-2753.2010.01516.x

[34] Mihalopoulos C , Harris M, Henry L, Harrigan S, McGorry P. Is early intervention in psychosis cost-effective over the long term? Schizophr Bull. 2009;35(5):909–918. 19509308 10.1093/schbul/sbp054 PMC2728818

[35] Chan SKW , Chan SWY, Pang HH, et al. Association of an early intervention service for psychosis with suicide rate among patients with first-episode schizophrenia-spectrum disorders. Jama Psychiatry. 2018;75(5):458–464. 29617517 10.1001/jamapsychiatry.2018.0185 PMC6145768

[36] Lambert M , Schottle D, Ruppelt F, et al. Early detection and integrated care for adolescents and young adults with psychotic disorders: the ACCESS III study. Acta Psychiatr Scand. 2017;136(2):188–200. 28589683 10.1111/acps.12762

[37] Keating D , McWilliams S, Boland F, et al. Prescribing pattern of antipsychotic medication for first-episode psychosis: a retrospective cohort study. BMJ Open. 2021;11(1):e040387. 10.1136/bmjopen-2020-040387 PMC7852941 33518516

[38] Chan SKW , Chau EHS, Hui CLM, Chang WC, Lee EHM, Chen EYH. Long term effect of early intervention service on duration of untreated psychosis in youth and adult population in Hong Kong. Early Interv Psychiatry. 2018;12(3):331–338. 26801970 10.1111/eip.12313

[39] Srihari VH , Tek C, Kucukgoncu S, et al. First-episode services for psychotic disorders in the us public sector: a pragmatic randomized controlled trial. Psychiatr Serv (Washington, D.C.). 2015;66(7):705–712. 10.1176/appi.ps.201400236 PMC4490067 25639994

[40] Chong SA , Mythily S, Verma S. Reducing the duration of untreated psychosis and changing help-seeking behaviour in Singapore. Soc Psychiatry Psychiatr Epidemiol. 2005;40(8):619–621. 16091855 10.1007/s00127-005-0948-4

[41] Malla A , Norman R, Scholten D, Manchanda R, McLean T. A community intervention for early identification of first episode psychosis—impact on duration of untreated psychosis (DUP) and patient characteristics. Soc Psychiatry Psychiatr Epidemiol. 2005;40(5):337–344. 15902403 10.1007/s00127-005-0901-6

[42] Ferrara M , Guloksuz S, Li F, et al. Parsing the impact of early detection on duration of untreated psychosis (DUP): applying quantile regression to data from the Scandinavian TIPS study. Schizophr Res. 2019;210:128–134. 31204063 10.1016/j.schres.2019.05.035

[43] Srihari V , Guloksuz S, Li F, et al. Mindmap: a population-based approach to early detection of psychosis in the United States. Paper presented at: International Congress on Schizophrenia Research, 2017.

[44] Connor C , Birchwood M, Freemantle N, et al. Don’t turn your back on the symptoms of psychosis: the results of a proof-of-principle, quasi-experimental intervention to reduce duration of untreated psychosis. Bmc Psychiatry. 2016;16:16. 27145865 10.1186/s12888-016-0816-7 PMC4855493

[45] Larsen TK , Melle I, Friis S, et al. One-year effect of changing duration of untreated psychosis in a single catchment area. Br J Psychiatry. 2007;191:S128–S132. 10.1192/bjp.191.51.s128 18055929

[46] Melle I , Larsen TK, Haahr U, et al. Prevention of negative symptom psychopathologies in first-episode schizophrenia. Arch Gen Psychiatry. 2008;65(6):634–640. 18519821 10.1001/archpsyc.65.6.634

[47] Browne J , Penn DL, Bauer DJ, et al. Perceived autonomy support in the NIMH RAISE early treatment program. Psychiatr Serv (Washington, D.C.). 2017;68(9):916–922. 10.1176/appi.ps.201600480 PMC12926809 28566027

[48] Bertelsen M , Jeppesen P, Petersen L, et al. Five-year follow-up of a randomized multicenter trial of intensive early intervention vs standard treatment for patients with a first episode of psychotic illness. Arch Gen Psychiatry. 2008;65(7):762–771. 18606949 10.1001/archpsyc.65.7.762

[49] Chan SKW , So HC, Hui CLM, et al. 10-Year outcome study of an early intervention program for psychosis compared with standard care service. Psychol Med. 2015;45(6):1181–1193. 25233868 10.1017/S0033291714002220

[50] Valmaggia LR , Byrne M, Day F, et al. Duration of untreated psychosis and need for admission in patients who engage with mental health services in the prodromal phase. Br J Psychiatry. 2015;207(2):130–134. 26045348 10.1192/bjp.bp.114.150623 PMC4655441

[51] Petrakis M , Penno S, Oxley J, Bloom H, Castle D. Early psychosis treatment in an integrated model within an adult mental health service. Eur Psychiatry. 2012;27(7):483–488. 21664801 10.1016/j.eurpsy.2011.03.004

[52] Melle I , Johannessen JO, Friis S, et al. Course and predictors of suicidality over the first two years of treatment in first-episode schizophrenia spectrum psychosis. Arch Suicide Res. 2010;14(2):158–170. 20455151 10.1080/13811111003704787

[53] Cullberg J , Levander S, Holmqvist R, Mattsson M, Wieselgren IM. One-year outcome in first episode psychosis patients in the Swedish Parachute project. Acta Psychiatr Scand. 2002;106(4):276–285. 12225494 10.1034/j.1600-0447.2002.02376.x

[54] McGorry PD , Edwards J, Mihalopoulos C, Harrigan SM, Jackson HJ. EPPIC: an evolving system of early detection and optimal management. Schizophr Bull. 1996;22(2):305–326. 8782288 10.1093/schbul/22.2.305

[55] Nishida A , Ando S, Yamasaki S, et al. A randomized controlled trial of comprehensive early intervention care in patients with first-episode psychosis in Japan: 1.5-year outcomes from the J-CAP study. J Psychiatr Res. 2018;102:136–141. 29653344 10.1016/j.jpsychires.2018.04.007

[56] Kane JM , Robinson DG, Schooler NR, et al. Comprehensive versus usual community care for first-episode psychosis: 2-year outcomes from the NIMH RAISE early treatment program. Am J Psychiatry. 2016;173(4):362–372. 26481174 10.1176/appi.ajp.2015.15050632 PMC4981493

[57] Nordentoft M , Albert N. Early intervention services are effective and must be defended. World Psychiatry. 2017;16(3):272–274. 28941094 10.1002/wps.20452 PMC5608812

[58] Addington J , Addington D. Social and cognitive functioning in psychosis. Schizophr Res. 2008;99(1–3):176–181. 17681756 10.1016/j.schres.2007.07.004

[59] Joa I , Johannessen JO, Auestad B, et al. The key to reducing duration of untreated first psychosis: information campaigns. Schizophr Bull. 2008;34(3):466–472. 17905788 10.1093/schbul/sbm095 PMC2632428

[60] Ly A , Tremblay GA, Beauchamp S. “What is the efficacy of specialised early intervention in mental health targeting simultaneously adolescents and young adults?” An HTA. Int J Technol Assess Health Care. 2019;35(2):134–140. 31017562 10.1017/S0266462319000084

[61] Padilla E , Molina J, Kamis D, et al. The efficacy of targeted health agents education to reduce the duration of untreated psychosis in a rural population. Schizophr Res. 2015;161(2–3):184–187. 25439394 10.1016/j.schres.2014.10.039 PMC4308442

[62] Qiu Y , Li L, Gan Z, et al. Factors related to duration of untreated psychosis of first episode schizophrenia spectrum disorder. Early Interv Psychiatry. 2019;13(3):555–561. 29164787 10.1111/eip.12519

[63] Conchon C , Sprüngli-Toffel E, Alameda L, et al. Improving pathways to care for patients at high psychosis risk in Switzerland: PsyYoung study protocol. J Clin Med. 2023;12(14):4642. 37510757 10.3390/jcm12144642 PMC10380609

[64] NHS-England. The National Collaborating Centre for Mental Health and the National Institute for Health and Care Excellence. Implementing the Early Intervention in Psychosis Access and Waiting Time Standard: Guidance. London, UK; 2016.

[65] Fusar-Poli P , Sullivan SA, Shah JL, Uhlhaas PJ. Improving the detection of individuals at clinical risk for psychosis in the community, primary and secondary care: an integrated evidence-based approach. Front Psychiatry. 2019;10:774. 31708822 10.3389/fpsyt.2019.00774 PMC6822017

[66] Davies C , Cipriani A, Ioannidis JPA, et al. Lack of evidence to favor specific preventive interventions in psychosis: a network meta-analysis. World Psychiatry. 2018;17(2):196–209. 29856551 10.1002/wps.20526 PMC5980552

[67] Fusar-Poli P , Salazar de Pablo G, Correll CU, et al. Prevention of psychosis: advances in detection, prognosis, and intervention. JAMA Psychiatry. 2020;77:755–765. 32159746 10.1001/jamapsychiatry.2019.4779

[68] Singh S. Early intervention in psychosis: much done, much more to do. World Psychiatry. 2017;16(3):276–277. 28941106 10.1002/wps.20455 PMC5608826

[69] Gebhardt S , Schmidt P, Remschmidt H, Hanke M, Theisen FM, König U. Effects of prodromal stage and untreated psychosis on subsequent psychopathology of schizophrenia: a path analysis. Psychopathology. 2019;52(5):304–315. 31734668 10.1159/000504202

[70] Galling B , Roldán A, Nielsen RE, et al. Type 2 diabetes mellitus in youth exposed to antipsychotics: a systematic review and meta-analysis. JAMA Psychiatry. 2016;73(3):247–259. 26792761 10.1001/jamapsychiatry.2015.2923

[71] Al-Dhaher Z , Kapoor S, Saito E, et al. Activating and tranquilizing effects of first-time treatment with aripiprazole, olanzapine, quetiapine, and risperidone in youth. J Child Adolesc Psychopharmacol. 2016;26(5):458–470. 27093218 10.1089/cap.2015.0141 PMC4931349

[72] Carbon M , Kapoor S, Sheridan E, et al. Neuromotor adverse effects in 342 youth during 12 weeks of naturalistic treatment with 5 second-generation antipsychotics. J Am Acad Child Adolesc Psychiatry. 2015;54(9):718–727.e3. 26299293 10.1016/j.jaac.2015.06.015 PMC10366711

[73] Højlund M , Haddad PM, Correll CU. Limitations in research on maintenance treatment for individuals with schizophrenia. JAMA Psychiatry. 2022;79(1):85–86. 34817573 10.1001/jamapsychiatry.2021.3452

[74] Højlund M , Kemp AF, Haddad PM, Neill JC, Correll CU. Standard versus reduced dose of antipsychotics for relapse prevention in multi-episode schizophrenia: a systematic review and meta-analysis of randomised controlled trials. Lancet Psychiatry. 2021;8(6):471–486. 34023019 10.1016/S2215-0366(21)00078-X

[75] Leucht S , Bauer S, Siafis S, et al. Examination of dosing of antipsychotic drugs for relapse prevention in patients with stable schizophrenia: a meta-analysis. JAMA Psychiatry. 2021;78(11):1238–1248. 34406325 10.1001/jamapsychiatry.2021.2130 PMC8374744

[76] Ostuzzi G , Vita G, Bertolini F, et al. Continuing, reducing, switching, or stopping antipsychotics in individuals with schizophrenia-spectrum disorders who are clinically stable: a systematic review and network meta-analysis. Lancet Psychiatry. 2022;9(8):614–624. 35753323 10.1016/S2215-0366(22)00158-4

[77] Taipale H , Tanskanen A, Luykx JJ, et al. Optimal doses of specific antipsychotics for relapse prevention in a nationwide cohort of patients with schizophrenia. Schizophr Bull. 2022;48(4):774–784. 35524479 10.1093/schbul/sbac039 PMC9212108

[78] Bailey T , Alvarez-Jimenez M, Garcia-Sanchez AM, Hulbert C, Barlow E, Bendall S. Childhood trauma is associated with severity of hallucinations and delusions in psychotic disorders: a systematic review and meta-analysis. Schizophr Bull. 2018;44(5):1111–1122. 29301025 10.1093/schbul/sbx161 PMC6101549

[79] Varese F , Smeets F, Drukker M, et al. Childhood adversities increase the risk of psychosis: a meta-analysis of patient-control, prospective- and cross-sectional cohort studies. Schizophr Bull. 2012;38(4):661–671. 22461484 10.1093/schbul/sbs050 PMC3406538

[80] Beards S , Gayer-Anderson C, Borges S, Dewey ME, Fisher HL, Morgan C. Life events and psychosis: a review and meta-analysis. Schizophr Bull. 2013;39(4):740–747. 23671196 10.1093/schbul/sbt065 PMC3686461

[81] Cullberg J , Mattsson M, Levander S, et al. Treatment costs and clinical outcome for first episode schizophrenia patients: a 3-year follow-up of the Swedish “Parachute Project” and Two Comparison Groups. Acta Psychiatr Scand. 2006;114(4):274–281. 16968365 10.1111/j.1600-0447.2006.00788.x

[82] Terry SF. Obama’s precision medicine initiative. Genet Test Mol Biomarkers. 2015;19(3):113–114. 25751403 10.1089/gtmb.2015.1563 PMC4361161

[83] Genetics Reference. What is precision medicine? https://ghr.nlm.nih.gov/primer/precisionmedicine/definition. Accessed June 6, 2023.

[84] Fusar-Poli P , Cappucciati M, Borgwardt S, et al. Heterogeneity of psychosis risk within individuals at clinical high risk a meta-analytical stratification. Jama Psychiatry. 2016;73(2):113–120. 26719911 10.1001/jamapsychiatry.2015.2324

[85] Salazar de Pablo G , Catalan A, Fusar-Poli P. Clinical validity of DSM-5 attenuated psychosis syndrome advances in diagnosis, prognosis, and treatment. Jama Psychiatry. 2020;77(3):311–320. 31746950 10.1001/jamapsychiatry.2019.3561

[86] Higgins J , Green S. Cochrane Handbook for Systematic Reviews of Interventions. Version 5.1.0; 2011. Cochrane.

[87] Salazar de Pablo G , Aymerich C, Guinart D, et al. What is the duration of untreated psychosis worldwide?—A meta-analysis of pooled mean and median time and regional trends and other correlates across 369 studies. Psychol Med. 2023;13:1–11. 10.1017/S0033291723003458 38087871

