# Supplementary material for: Duration of Untreated Psychosis and Outcomes in First-Episode Psychosis: Systematic Review and Meta-analysis of Early Detection and Intervention Strategies
Source: Schizophr Bull. 2024 Mar 16;50(4):771–83. doi: 10.1093/schbul/sbae017 (PMC11283197; doi:10.1093/schbul/sbae017)
Supplement: sbae017_suppl_Supplementary_Material [file sbae017_suppl_supplementary_material.docx]

**SUPPLEMENTARY MATERIAL**

**eTable 1, page 2-4:** PRISMA statement and checklist

**eTable 2, page 5-6:** Meta-analysis Of Observational Studies in Epidemiology **(**MOOSE) checklist

**eTable 3, page 7-8:** Definitions and instruments for mental health outcomes

**eTable 4, page 9-14:** Main characteristics of included studies

**eTable 5, page 15-16:** Meta-regression analyses between early detection outcomes and moderating factors

**eTable 6, page 17-18:** Meta-regression analyses between early intervention outcomes and moderating factors

**eFigures 1, page 19-21:** Forest plots meta-analytic outcomes of early detection strategies

**eFigures 2, page 22-24:** Forest plots meta-analytic outcomes of early intervention strategies

**eFigures 3, page 25-28:** Funnel plots meta-analytic outcomes of early detection strategies

**eFigures 4, page 29-33:** Funnel plots meta-analytic outcomes of early intervention strategies

**eFigure 5, page 34:** Other potential benefits of early detection and early Intervention strategies based on systematic review results

**eFigure 6, page 35:** Quality assessment results

**This supplementary material has been provided by the authors to give readers additional information about their work.**

**eTable 1: PRISMA statement and checklist**

| **Section and Topic** | **Item #** | **Checklist item** | **Location where item is reported** |
| --- | --- | --- | --- |
| **TITLE** | | |  |
| Title | 1 | Identify the report as a systematic review. | Title page |
| **ABSTRACT** | | |  |
| Abstract | 2 | See the PRISMA 2020 for Abstracts checklist. | Abstract page |
| **INTRODUCTION** | | |  |
| Rationale | 3 | Describe the rationale for the review in the context of existing knowledge. | Introduction |
| Objectives | 4 | Provide an explicit statement of the objective(s) or question(s) the review addresses. | Introduction |
| **METHODS** | | |  |
| Eligibility criteria | 5 | Specify the inclusion and exclusion criteria for the review and how studies were grouped for the syntheses. | Methods |
| Information sources | 6 | Specify all databases, registers, websites, organisations, reference lists and other sources searched or consulted to identify studies. Specify the date when each source was last searched or consulted. | Methods |
| Search strategy | 7 | Present the full search strategies for all databases, registers and websites, including any filters and limits used. | Methods |
| Selection process | 8 | Specify the methods used to decide whether a study met the inclusion criteria of the review, including how many reviewers screened each record and each report retrieved, whether they worked independently, and, if applicable, details of automation tools used in the process. | Methods |
| Data collection process | 9 | Specify the methods used to collect data from reports, including how many reviewers collected data from each report, whether they worked independently, any processes for obtaining or confirming data from study investigators, and if applicable, details of automation tools used in the process. | Methods |
| Data items | 10a | List and define all outcomes for which data were sought. Specify whether all results that were compatible with each outcome domain in each study were sought (e.g. for all measures, time points, analyses), and if not, the methods used to decide which results to collect. | Methods |
|  | 10b | List and define all other variables for which data were sought (e.g. participant and intervention characteristics, funding sources). Describe any assumptions made about any missing or unclear information. | Methods |
| Study risk of bias assessment | 11 | Specify the methods used to assess risk of bias in the included studies, including details of the tool(s) used, how many reviewers assessed each study and whether they worked independently, and if applicable, details of automation tools used in the process. | Methods |
| Effect measures | 12 | Specify for each outcome the effect measure(s) (e.g. risk ratio, mean difference) used in the synthesis or presentation of results. | Methods |
| Synthesis methods | 13a | Describe the processes used to decide which studies were eligible for each synthesis (e.g. tabulating the study intervention characteristics and comparing against the planned groups for each synthesis (item #5)). | Methods |
|  | 13b | Describe any methods required to prepare the data for presentation or synthesis, such as handling of missing summary statistics, or data conversions. | Methods |
|  | 13c | Describe any methods used to tabulate or visually display the results of individual studies and syntheses. | Methods |
|  | 13d | Describe any methods used to synthesise results and provide a rationale for the choice(s). If meta-analysis was performed, describe the model(s), method(s) to identify the presence and extent of statistical heterogeneity, and software package(s) used. | Methods |
|  | 13e | Describe any methods used to explore possible causes of heterogeneity among study results (e.g. subgroup analysis, meta-regression). | Methods |
|  | 13f | Describe any sensitivity analyses conducted to assess the robustness of the synthesised results. | Methods |
| Reporting bias assessment | 14 | Describe any methods used to assess risk of bias due to missing results in a synthesis (arising from reporting biases). | Methods |
| Certainty assessment | 15 | Describe any methods used to assess certainty (or confidence) in the body of evidence for an outcome. | Methods |
| **RESULTS** | | |  |
| Study selection | 16a | Describe the results of the search and selection process, from the number of records identified in the search to the number of studies included in the review, ideally using a flow diagram. | Results, Figure 1 |
|  | 16b | Cite studies that might appear to meet the inclusion criteria, apart from those which were excluded, and explain why they were excluded. | NA |
| Study characteristics | 17 | Cite each included study and present its characteristics. | eTable IV |
| Risk of bias in studies | 18 | Present assessments of risk of bias for each included study. | eTable IV |
| Results of individual studies | 19 | For all outcomes, present, for each study: (a) summary statistics for each group (where appropriate) and (b) an effect estimate and its precision (e.g. confidence/credible interval), ideally using structured tables or plots. | Results, tables |
| Results of syntheses | 20a | For each synthesis, briefly summarise the characteristics and risk of bias among contributing studies. | Results |
|  | 20b | Present results of all statistical syntheses conducted. If meta-analysis was done, present for each the summary estimate and its precision (e.g. confidence/credible interval) and measures of statistical heterogeneity. If comparing groups, describe the direction of the effect. | Results |
|  | 20c | Present results of all investigations of possible causes of heterogeneity among study results. | Results, eTable V-VI |
|  | 20d | Present results of all sensitivity analyses conducted to assess the robustness of the synthesised results. | Results, eTable V-VI |
| Reporting biases | 21 | Present assessments of risk of bias due to missing results (arising from reporting biases) for each synthesis assessed. | Results |
| Certainty of evidence | 22 | Present assessments of certainty (or confidence) in the body of evidence for each outcome assessed. | Results |
| **DISCUSSION** | | |  |
| Discussion | 23a | Provide a general interpretation of the results in the context of other evidence. | Discussion |
|  | 23b | Discuss any limitations of the evidence included in the review. | Discussion, |
|  | 23c | Discuss any limitations of the review processes used. | Discussion, |
|  | 23d | Discuss the implications of the results for practice, policy, and future research. | Discussion |
| **OTHER INFORMATION** | | |  |
| Registration and protocol | 24a | Provide registration information for the review, including a register name and registration number, or state that the review was not registered. | Methods |
|  | 24b | Indicate where the review protocol can be accessed or state that a protocol was not prepared. | Methods |
|  | 24c | Describe and explain any amendments to the information provided at registration or in the protocol. | NA |
| Support | 25 | Describe sources of financial or non-financial support for the review and the role of the funders or sponsors in the review. | Discussion |
| Competing interests | 26 | Declare any competing interests of review authors. | Discussion |
| Availability of data, code and other materials | 27 | Report which of the following are publicly available and where they can be found: template data collection forms; data extracted from included studies; data used for all analyses; analytic code; any other materials used in the review. | Discussion |

**eTable 2: Meta-analysis Of Observational Studies in Epidemiology (MOOSE) checklist**^1^

| **Criteria** | | **Brief description of how the criteria were handled in the meta-analysis** |
| --- | --- | --- |
| **Reporting of background should include** | |  |
| √ | Problem definition | No meta-analysis has analyzed the efficacy of early detection and early intervention strategies altering DUP on real-world outcomes. |
| √ | Hypothesis statement | We hypothesised that early detection would have a modest impact on real-world outcomes.  We hypothesised that early intervention would have a positive impact on real-world outcomes |
| √ | Description of study outcomes | Study outcomes are defined in the main text and first table. |
| √ | Type of exposure or intervention used | Interventions were based on early intervention by multidisciplinary teams of mental health professionals providing multimodal psychosocial and psychopharmacological interventions. |
| √ | Type of study designs used | Intervention studies (randomised or non-randomised) with a different control group. |
| √ | Study population | Subjects with an early onset psychosis. |
| **Reporting of search strategy should include** | |  |
| √ | Qualifications of searchers | Qualifications of researchers were specified.  The credentials of the investigators are indicated in the author list and in the acknowledgements. |
| √ | Search strategy, including time period included in the synthesis and keywords | We performed a multi-step literature search (keywords in the methods section) from inception until February 1, 2023. |
| √ | Databases and registries searched | PubMed Web of Science database (Web of Science Core Collection, BIOSIS Citation Index, KCI-Korean Journal Database, MEDLINE, Russian Science Citation Index, and SciELO Citation Index). |
| √ | Use of hand searching | We hand-searched bibliographies of retrieved papers for additional references. |
| √ | List of citations located and those excluded, including justifications | Details of the literature search process can be found in the results section and PRISMA flowchart. |
| √ | Method of addressing articles published in languages other than English | Articles in any language were selected. We contacted native speakers to extract information in other languages. |
| √ | Method of handling abstracts and unpublished studies | Original individual studies; abstracts, and conference proceedings were included. Reviews were excluded. |
| √ | Description of any contact with authors | We did not contact corresponding authors to gather additional data. |
| **Reporting of methods should include** | |  |
| √ | Description of relevance or appropriateness of studies.  to be tested | Detailed inclusion and exclusion criteria were described in the methods section. |
| √ | Rationale for the selection and coding of data | Data extracted from each of the studies were relevant to the population characteristics, study design, and the studies outcomes. |
| √ | Assessment of confounding | We conducted meta-analytical regressions whenever four or more studies were available to estimate the association between efficacy of the intervention on each of the outcomes and (i) program continent (ii) FEP diagnosis (iii) control content, (iv) age (v), sex (vi), DUP ( (vi) duration of the intervention -only for intervention analysis- and (vii) quality of the study (weak, moderate, strong). |
| √ | Assessment of study quality | We assessed the quality of the studies using “Effective Public Health Practice Project” (EPHPP) |
| √ | Assessment of heterogeneity | Heterogeneity was assessed with the Q statistics. The proportion of the total variability in the effect size estimates was evaluated with the I^2^ index. |
| √ | Description of statistical methods in sufficient detail to be replicated | The effect size was estimated when three or more studies were available by calculating the Hedges' g. A random-effects meta-analysis was used. More details are described in the methods section. |
| √ | Provision of appropriate tables and graphics | We provided several tables and graphs in the main text and supplementary section to describe the literature search and its results. |
| **Reporting of results should include** | |  |
| √ | Table summarising individual study estimates and the overall estimate | We summarised individual study estimates and overall estimates in the text. |
| √ | Table giving descriptive information for each study included | We presented descriptive information for each study in the tables and as supplementary material. |
| √ | Results of sensitivity testing | Additional analyses were conducted as specified in the manuscript. |
| √ | Indication of statistical uncertainty of findings | We reported this in the results section. |
| **Reporting of discussion should include** | |  |
| √ | Quantitative assessment of bias | The presence of publication bias in the results was assessed informally by visually inspecting funnel plots, complemented by the “trim and fill” method to investigate the effects of any publication bias detected. |
| √ | Justification for exclusion | We excluded studies based on the rationale of the meta-analysis as stated in the manuscript. |
| √ | Assessment of quality of included studies | The quality of the studies was assessed as detailed in the main and the supplementary section of the manuscript. |
| **Reporting of conclusions should include** | |  |
| √ | Consideration of alternative explanations for observed results | This point has been addressed in the discussion section. |
| √ | Generalisation of the conclusions | This point has been addressed in the discussion section. |
| √ | Guidelines for future research | This point has been addressed in the discussion section. |
| √ | Disclosure of funding source | This point has been addressed at the end of the manuscript. |

**eTable 3: Definitions and instruments for mental health outcomes**

| **Outcome** | **Definition/ Scale Used** |
| --- | --- |
| **Positive symptoms** | The Positive and Negative Syndrome Scale (PANSS)^2^  The Scale for the Assessment of Positive Symptoms (SAPS)^3^  The Brief Psychiatric Rating Scale (BPRS)^4^ |
| **Negative symptoms** | The Positive and Negative Syndrome Scale (PANSS)^2^  The Scale for the Assessment of Negative Symptoms (SANS)^5^ |
| **Total symptoms** | The Positive and Negative Syndrome Scale (PANSS)^2^  The Brief Psychiatric Rating Scale (BPRS) ^4^ |
| **Depressive symptoms** | Hamilton Rating Scale for Depression (HAM-D)^6^  Calgary Depression Scale for Schizophrenia (CDSS)^7^  Beck Depression Inventory (BDI)^8^ |
| **Functioning** | Global Assessment of Functioning (GAF)^9^  Social and Occupational Functioning Assessment Scale (SOFAS)^10^  Global Functioning: Role (GFR); Global Functioning: Social (GFS)^11,12^ |
| **Quality of Life** | Quality of Life Scale (QLS)^13^  Short Form Health Survey (SF-12)^14^  World Health Organization Quality of Life (WHO-QoL)^15^ |
| **Remission** | Percentage of patients who met study-defined definition of remission, indicating symptom stability and/or minimum symptom severity^16^ |
| **Recovery** | Percentage of patients who met the study-defined definition of remission, indicating symptom stability/minimum severity plus improved social/ educational/ vocational attainment^16^ |
| **Relapse** | Percentage of patients who met the study-defined definition of relapse^16^ |
| **Employment** | Percentage of patients with a part-time or full-time work |
| **Mean number of admissions** | Mean number of psychiatric admissions per patient for any reason and independent of length of hospitalization^16^ |

Positive symptoms were evaluated with the Positive and Negative Syndrome Scale (PANSS)^2^, the Scale for the Assessment of Positive Symptoms (SAPS)^3^ or the Brief Psychiatric Rating Scale (BPRS)^4^.

Negative symptoms were evaluated with the Positive and Negative Syndrome Scale (PANSS)^2^ or the Scale for the Assessment of Negative Symptoms (SANS)^5^.

Total symptoms were evaluated with the Positive and Negative Syndrome Scale (PANSS)^2^ or the Brief Psychiatric Rating Scale (BPRS)^4^.

Depressive symptoms were evaluated with the Hamilton Rating Scale for Depression (HAM-D)^6^, the Calgary Depression Scale for Schizophrenia (CDSS)^7^ or the Beck Depression Inventory (BDI)^8^.

Functioning was evaluated with the Global Assessment of Functioning (GAF)^9^, the Social and Occupational Functioning Assessment Scale (SOFAS)^10^ or the Global Functioning: Role (GFR); Global Functioning: Social (GFS)^11,12^.

Quality of Life was evaluated with the Quality of Life Scale (QLS)^13^, the Short Form Health Survey (SF-12)^14^ or World Health Organization Quality of Life (WHO-QoL)^15^

| **Definition DUP** | | |
| --- | --- | --- |
| Barajas Velez, 2016^17^ | N.a. |  |
| Bertelsen, 2008^18^ | Duration of untreated psychosis as per IRAOS (Interview for Retrospective Assessment of Onset of Schizophrenia). |  |
| Browne, 2017^19^ | N.a. |  |
| Cassidy, 2008^20^ | Period between the time of first onset of psychotic symptoms to the time of adequate treatment with antipsychotics. |  |
| Chan, 2015^21^ | Time between first onset of positive symptoms to presentation to the mental health service. |  |
| Chan, 2018^22^ | Time interval between the first appearance of psychotic symptoms and the initiation of treatment |  |
| Chan, 2018^23^ | Period between the first appearance of psychotic symptoms and the use of successful psychiatric treatment. |  |
| Chong, 2006^24^ | Time from the onset of hallucinations and/or delusions, disorganised thinking and/or behaviour to the time of appropriate treatment. |  |
| Chong, 2005^25^ | Time between the onset of psychotic symptoms and the time when a definitive diagnosis and treatment are established. |  |
| Connor, 2016^26^ | Delay between the onset of a first episode of psychosis and receipt of treatment. |  |
| Craig, 2004^27^ | Period from the first onset of positive symptoms to initial contact with psychiatric services in patients for whom this was their first contact. |  |
| Cullberg, 2002^28^ | Time from earliest date of experienced or observed psychotic symptoms to treatment. |  |
| Ferrara, 2019^29^ | Period of time between the emergence of psychotic symptoms and initiation of appropriate clinical treatment. |  |
| Hegelstad, 2012^30^ | Time between the onset of the first psychotic episode and the start of first adequate treatment. |  |
| Joa, 2008^31^ | Time from onset of psychosis until the start of adequate treatment. |  |
| Kane, 2016^32^ | Time from the onset of psychosis to the start of treatment. |  |
| Keating, 2021^33^ | Interval between first experience of psychotic symptoms and presentation to the psychiatric services for initiation of treatment. |  |
| Krstev, 2004^34^ | Time between the onset of psychosis and the commencement of treatment. |  |
| Lambert, 2017^35^ | Duration of untreated psychosis as per Royal Park Multidiagnostic Instrument for Psychosis. |  |
| Larsen, 2007^36^ | Time from onset of psychosis until the start of adequate treatment. |  |
| Lloyd-Evans, 2015^37^ | Duration from first psychotic symptom to first contact with CIEIS. |  |
| Malla, 2014^38^ | Time between the onset of the current psychotic episode and adequate treatment with antipsychotic medication. |  |
| Malla, 2005^39^ | Time from the time of the first ever onset of psychotic symptoms and adequate treatment. |  |
| McGorry, 1996^40^ | N.a. |  |
| Melle, 2004^41^; Melle, 2008^42^; Melle, 2010^43^ | Time from onset of psychosis until the start of adequate treatment. |  |
| Mihalopoulos, 2009^44^;Nishida, 2018^45^; Petrakis, 2012^46^ | N.a. |  |
| Srihari, 2015^47^ | Time between onset of psychosis defined by the Symptom Onset in Schizophrenia scale and initiation of antipsychotic treatment. |  |
| Srihari, 2017^48^ | Time between the onset of psychosis and initiation of antipsychotic treatment |  |
| Valmaggia, 2015^49^ | Period between the onset of frank psychosis and the initiation of its treatment |  |

**eTable 4**: **Main characteristics of included studies**

| **Author, year** | **Program; Country** | **Sample size:**  **total (INT; CTRL)** | **Mean age** | **% males** | **DUP INT (Mean± SD or Median)** ^a^ | **DUP CTRL (Mean± SD or Median)** ^a^ | **QA** | **Key findings** |
| --- | --- | --- | --- | --- | --- | --- | --- | --- |
| Barajas Velez, 2016^17^ | PAE-TPI, Spain | 191 (133;58) | N.a. | N.a. | 5.7 | 12.8 | Weak | After 15 months, waiting time decreased in the intervention group (11.2 vs. 40.8 days; p=0.007). |
| Bertelsen, 2008^18^ | OPUS, Denmark | 547 (275;272) | 26.6 | 59.0 | 46 | 53 | Moderate | Psychotic and negative dimensions were not better in those individuals who had been in the early intervention group after five years (p>0.05). Less individuals in the early intervention group were living in supported housing (4% vs 10%, p=0.02). They were hospitalized fewer days (149 vs 193 days, p=0.05). |
| Browne, 2017^19^ | RAISE,  USA | 404 (223;181) | 23.1 | 72.5 | 178±248.7 | 211.4±277.5 | Weak | Perceived autonomy increased in the intervention group after 24 months of treatment (p<0.01). |
| Cassidy, 2008^20^ | PEPP, Canada | 194 (106;88) | N.a. | N.a. | 22.8 | 24.5 | Weak | Only for individuals with schizophrenia spectrum psychoses, median DUP decreased (p=0.020). |
| Chan, 2015^21^ | EASY, China | 209 (102;107) | 21.8 | 50.7 | 36.3±47.7 | 40.7±47.5 | Weak | Individuals in the intervention group had fewer (OR=1.56, p<0.0001) and shorter (OR=1.29, p=0.04), hospitalizations and longer employment periods (OR=0.28, p<0.0001). They also had reduced suicide rates (p = 0.037) and less suicide attempts (p=0.001) over 10 years. |
| Chan, 2018^22^ | EASY, China | 601 (479;122) | 33 | 45.3 | 13.3 | 17-25.7^b^ | Moderate | DUP was particularly reduced in the early intervention group for those individuals without a family history of mental illness in the adult individuals (p=0.01). It also was also reduced in youth individuals with a gradual onset (p=0.01). |
| Chan, 2018^23^ | EASY, China | 1234 (617;617) | 21.2 | 51.8 | 13.1 | 8.7 | Weak | The suicide rates decreased in the intervention group (7.5% vs 4.4%, p=0.02) compared to the control group. The number of suicide attempts while psychosis was untreated (p=0.009) was associated with early suicide. Premorbid occupational impairment, (p=0.02) number of relapses in the first 3 years (p=0.04), and poor adherence in the first 3 years (p=0.07) were associated with suicide after 4-12 years. |
| Chong, 2006^24^ | EPIP, Singapore | 551 (483;68) | 28.4 | N.a. | 16 | 24 | Weak | The rates of antipsychotic polypharmacy, prolonged use of benzodiazepines and anticholinergic medication in the intervention group were lower. The dose of antipsychotics was also lower in the intervention group at baseline: (p=0.01) and at the third month (p=0.004). 87.5% of EPIP patients were responders by the third month. |
| Chong, 2005^25^ | EPIP, Singapore | 394 (287;107) | 30.9 | 55.4 | 53.2±104 | 128±237.2 | Moderate | The median DUP was reduced from 12 to median of 4 months (p=0.002). Police referrals decreased (p=0.001) and self and family referrals increased (p=0.04). |
| Connor, 2016^26^ | EDEN/Youthspace, UK | 151 (77;74) | 22.0 | 64.9 | 14.8±68.9 | 30.8±65.6 | Weak | In the intervention group, DUP (p=0.01), help-seeking delay (p=0.01) and delay in reaching the mental health services (p=0.003) decreased compared to the control group. |
| Craig, 2004^27^ | LEO, UK | 144 (71;73) | 26.3 | 64.6 | 42±68.8 | 30.4±42.8 | Strong | Patients in the intervention group were more likely to recover at 18 months (p=0.035), less likely to relapse (p=0.042) and were readmitted fewer times during follow up (p=0.01). After adjusting for baseline differences in sex, previous psychotic episodes, and ethnic minority group, only the difference in total number of readmissions during follow up remained statistically significant (p=0.03). |
| Cullberg, 2002^28^ | Parachute Project, Sweden | 388 (253;135) | 28.9 | 51.8 | 21 | 12 | Weak | Psychiatric in-patient care (p<0.01) and prescription of neuroleptic medication (p<0.05) was lower in the intervention compared to the comparison group. Satisfaction was high in the intervention group (3.9/5 for patients and 4/5 for relatives). |
| Ferrara, 2019^29^ | TIPS, Norway/ Denmark | 281 (141;140) | 28.6 | 59.1 | 41.6±122.8 | 56.5±118.3 | Moderate | No differences were found in premorbid functioning between the groups (p>0.05). Male gender was associated with longer DUP. Early detection campaign had a bigger effect on DUP for the stratified group with the longer (median=26 weeks) compared to the group with a shorter (median=3weeks) DUP (p=0.009). Male gender (p=0.01) and single status (p=0.04) predicted longer DUP in the group with the longer DUP (median=26 weeks). |
| Hegelstad, 2012^30^ | TIPS, Norway | 174 (101;73) | 28.4 | 55.4 | 4 | 13 | Moderate | Higher % of early-detection patients had recovered (OR=2.5, p=0.017), and had a full-time work (OR=3.1, p=0.007) at the 10-year follow-up compared to the control group. |
| Joa, 2008^31^ | TIPS, Norway^c^ | 183 (108;75) | 25.2 | 61.7 | 26±58.6 | 105±275.8 | Moderate | DUP increased in the comparison group when the information campaign stopped (p<0.005). They intervention group with the information campaign had more positive symptoms, total symptoms, and better functioning (p<0.05) |
| Kane, 2016^32^ | RAISE, USA | 404 (223; 181) | 23.1 | 72.5 | 178.9±248.7 | 211.4±277.5 | Moderate | Total psychopathology (p<0.02), and depression (p<0.05), improved in the intervention group after 24 months compared to the control group. The adherence to the treatment was higher (p<0.004) and they were more likely to have received mental health outpatient services (p=0.013). Those individuals with a shorter DUP (<74 weeks) had a greater improvement in quality of life (p=0.003) and psychopathology (p=0.043) compared with those with longer DUP. |
| Keating, 2021^33^ | DETECT, Ireland | 490 (319; 171) | 31.1 | 58.8 | 5 | 3 | Moderate | The proportion of SGAs increased from 32.2% in the control to 90.4-92.4% in the intervention group.  First-generation antipsychotics were prescribed  for 65.1% of the control group compared with 4.3-5.5% of the intervention group. 2.7% in the control group and 3.3-3.9% in the intervention group were not prescribed antipsychotic medication. |
| Krstev, 2004^34^ | EPPIC, Australia | 98 (40;58) | 22.2 | 69.4 | 44.8±79.8 | 36.3±54.2 | Weak | There were differences between the groups in the distribution of DUP (short DUP<1 year; long DUP 1-3 years; very long >3 years) (p = 0.005), being cases with very long DUP detected in the intervention group (15.0%) more compared to the control group (3.4%). |
| Lambert, 2017^35^ | ACCESS-III/EDIC, Germany | 225 (120;105) | 21.2 | 59.1 | 16.4 | 29.4 | Weak | Symptomatic and functional remission was more frequent in the intervention group (48.9%) compared to the control group (15.2%) (p<0.001). Receiving the intervention (OR=6.8) and an older age (OR=1.1) predicted remission. |
| Larsen, 2007^36^ | TIPS, Norway | 76 (41;35) | 25.8 | 52.8 | 6 | 26 | Moderate | Less patients in the early detection group were hospitalized compared to the control group (p=0.001) after one year compared to the historical control group. Individuals in the early detection group had less positive (p=0.003), negative (p=0.0001) and general (p=0.0001) symptoms. They also had more friends (p=0.02) after one year. No differences were found in clinical course or employment after 12 months. |
| Lloyd-Evans, 2015^37^ | CIEIS, UK | 180 (110;70) | 24.3 | 68.9 | 42.1±66.9 | 56.6±106.1 | Weak | Barriers to referrals included uncertainty about the signs of early psychosis, disengagement by young people when becoming unwell, and worries about stigma or coercive treatment. Satisfaction was high with workshops: 97% staff thought the information provided was useful to recognize psychosis and they would recommend the intervention. After the intervention knowledge about psychosis improved, stigmatizing attitudes decreased and attitudes to mental health services improved (all p<0.001). Pathway to care in the intervention and control group differed (p=0.003), reaching more individuals their care without having previous contact with mental health services (17%) in the intervention compared to the control group (3%) the year before. |
| Malla, 2014^38^ | PEPP, Canada | 295 (159;136) | 21.9 | 68.9 | 43.5±71.6 | 43.5±61.7 | Weak | There was an increase in the number of FEP individuals referred from hospitals other than the parent institute in the intervention group. There was an increase in the proportion of affective psychosis (p<0.05) and patients with low socioeconomic status (SES) (p<0.01) in the intervention group compared to the control group. |
| Malla, 2005^39^ | PEPP, Canada | 188 (100;88) | 25.5 | 78.2 | 24.3 | 21.9 | Strong | No differences in referrals were found before and after the community early detection program was established. Patients entering treatment after the community early detection program had more total symptoms (p<0.01) and more positive symptoms (p< 0.001), but not negative symptoms, depression or anxiety (p>0.05). |
| McGorry, 1996^40^ | EPPIC, Australia | 102 (51;51) | 22.2 | 64.7 | 27.3±69 | 33.8±100.4 | Weak | The intervention group had less admissions (p<0.01) and inpatient bed days (p <0.001). Maximum initial dosage of antipsychotic was lower (p <0.001). No differences in positive or negative symptoms were found after 12 months (p>0.05) between the groups. Quality of life was higher in the intervention group (p=0.009). |
| Melle, 2004^41^ | TIPS, Norway | 281 (141;140) | 28.6 | 59.0 | 5 | 16 | Moderate | In the early detection area, individuals detected in the intervention group had less severe positive, negative and general symptoms as well as better functioning (all *p <*0.01). After three months, individuals in the intervention group had less severe negative symptoms *(p<*0.01), general symptoms (p*<*0.05) and functioning (p*<*0.05) but not positive symptoms (p>0.05). |
| Melle, 2008^42^ | TIPS Norway | 231 (118;113) | 28.6 | 58.3 | 5 | 16 | Strong | After two year, in the intervention group, negative (p<0.001), cognitive (p<0.001) and depressive (p<0.001) symptoms (measured all by PANSS) improved compared to the control group. There were no statistically significant differences between groups in positive symptoms, number of patients in remission or relapse, number of relapse or functioning (p>0.05). |
| Melle, 2010^43^ | TIPS, Norway | 231 (118;113) | 28.5 | 58.4 | 5 | 16 | Moderate | Suicidality was lower at baseline (p<0.001), 1-year (p=0.001) and 2-year (p=0.03) follow up in the intervention compared to the control group. |
| Mihalopoulos, 2009^44^ | EPPIC, Australia | 65 (32;33) | 22.5 | 61.5 | 5 | 16 | Moderate | 8 years after the initial intervention, individuals in the intervention group had less severe positive symptoms (p=0.007), were more frequently in remission (p=0.008) and had a better course of illness (p=0.011) than the control group. Total mean mental health service costs were lower (p=0.01) in the intervention compared to the control group. |
| Nishida, 2018^45^ | J-CAP, Japan | 77 (40;37) | 23.0 | 55.8 | 60,4±47,2 | 74.8±64.4 | Weak | The intervention group had higher clinical remission rates (OR=6.3, p=0.045) and lower dropout rates (OR=0.038, p=0.045) compared to the control group. No differences in functioning were found (p=0.195). Family satisfaction, initially higher in the intervention group (OR=3.69, p=0.042), did not remain significant after adjusting for baseline characteristics (p=0.697). |
| Petrakis, 2012^46^ | EPP, Australia | 122 (60;62) | 29.0 | 55.7 | 12 | 12 | Weak | In the intervention group, less patients had one or more hospital admission (p=0.004), and the number of admissions was lower (p=0.023). The locked units were less used (p=0.002). No significant differences in the rates of police involvement (p=0.130) in admission and use of seclusion (p=0.368) were found between the intervention and the control group. |
| Srihari, 2015^47^ | STEP, USA | 117 (60;57) | 22.5 | 81.5 | 40±64 | 40±52 | Weak | After one year, individuals in the intervention group had fewer inpatient admissions (RR=1.38, p=0.014), less hospitalizations (p=0.023); and less bed-days (p=0.046). Vocational engagement (RR=1.40, p=0.04) was also higher in the intervention group than the control group. |
| Srihari, 2017^48^ | STEP, USA | 75 (53;22) | N.a. | N.a. | 48 | 118 | Moderate | No differences between the groups in help-seeking attempts were found. |
| Valmaggia, 2015^49^ | OASIS, UK^d^ | 190 (43;147) | 24.1 | 72.1 | 1.5±1.7 | 52.3±148.7 | Moderate | Compared to individuals in the control group, individuals treated in an ARMS service, had less hospital admissions (p=0.01) and less admissions under the Mental Health Act (p=0.01). No group differences in the duration of hospital admissions were found (p=0.140). Patients treated in the ARMS service had higher employment rates (p<0.001). They were less likely from an ethnic minority (p=0.017). |

^a^ If only one number is provided it represents median DUP; ^b^ Median age of the youth and adult group respectively; ^c^ The comparison group received an early intervention without information campaign.; ^d^The comparison group received an early intervention once FEP was established. The intervention group worked with ARMS individuals.

CIEIS: Camden and Islington Early Intervention Service; CTRL: Control; DETECT: Dublin and East Treatment and Early Care Team; DUP: Duration of untreated psychosis; EDIC: Early detection plus integrated care; EPIP: Early Psychosis Intervention Programme; EPPIC: Early Psychosis Prevention and Intervention Centre; INT: Intervention; J-CAP: Japanese Comprehensive Approach for First-episode Psychosis; LEO: Lambeth Early Onset; OASIS: Outreach and Support in South London; PAE-TPI: Intensive Early Intervention Programme for Incipient Psychosis; PEPP: Prevention and Early intervention in Psychosis Program; QA: Quality assessment; RAISE; Recovery After an Initial Schizophrenia Episode; SGA: Second generation antipsychotic; STEP: Specialized Treatment Early in Psychosis TIPS: Early Treatment and Intervention in Psychosis study.

**eTable 5: Meta-regression analyses between early detection outcomes and moderating factors**

| **Outcome** |  | **No. of**  **Studies** | **β Coefficient** | **SE** | **95% CI** | | **Z-Value** | **P value** |
| --- | --- | --- | --- | --- | --- | --- | --- | --- |
| **DUP** | Continent | 14 | -0.007 | 0.139 | -0.279 | 0.264 | -0.76 | 0.445 |
|  | % Affective psychosis | 12 | -0.001 | 0.002 | -0.006 | 0.003 | -0.597 | 0.551 |
|  | Control content | 14 | -0.140 | 0.110 | -0.357 | 0.076 | -1.271 | 0.204 |
|  | Mean age | 14 | -0.552 | 0.472 | -0.008 | 0.067 | 1.544 | 0.123 |
|  | Sex | 14 | -0.006 | 0.008 | -0.021 | 0,009 | -0.814 | 0.416 |
|  | Quality of the study | 14 | 0.060 | 0.095 | -0.126 | 0.246 | 0.631 | 0.528 |
| **Positive symptoms** | **DUP** | **8** | **0.0034** | **0.005** | **-0.007** | **0.014** | **0.617** | **0.537** |
|  | Continent | 8 | 0.171 | 0.160 | -0.142 | 0.484 | 1.072 | 0.284 |
|  | % Affective psychosis | 7 | 1.227 | 1.157 | -1.041 | 3.495 | 1.061 | 0.289 |
|  | Control content | 8 | 0.139 | 0.239 | -0.544 | 0.823 | 0.399 | 0.690 |
|  | Mean age | 8 | 0.041 | 0.041 | -0.040 | 0.122 | 0.985 | 0.324 |
|  | Sex | 8 | -0.010 | 0.013 | -0.037 | 0.016 | -0.751 | 0.452 |
|  | Quality of the study | 8 | -0.164 | 0.183 | -0.522 | 0.193 | -0.901 | 0.368 |
| **Negative symptoms** | **DUP** | **10** | **0.001** | **0.004** | **-0.007** | **0.008** | **0.143** | **0.997** |
|  | Continent | 10 | 0.201 | 0.300 | -0.387 | 0.790 | 0.671 | 0.502 |
|  | % Affective psychosis | 9 | 1.325 | 0.868 | -0.376 | 3.027 | 1.527 | 0.127 |
|  | Control content | 10 | 0.264 | 0.232 | -0.190 | 0.718 | 1.140 | 0.254 |
|  | Mean age | 10 | 0.038 | 0.032 | -0.023 | 0.100 | 1.212 | 0.226 |
|  | Sex | 10 | 0.010 | 0.008 | -0.025 | 0.005 | -1.270 | 0.204 |
|  | Quality of the study | 10 | 0.093 | 0.347 | -0.587 | 0.774 | 0.269 | 0.788 |
| **Total psychopathology^a^** | **DUP** | **7** | **0.001** | **0.008** | **-0.0147** | **0.018** | **0.190** | **0.849** |
|  | Continent | 7 | 0.172 | 0.346 | -0.506 | 0.850 | 0.498 | 0.619 |
|  | % Affective psychosis | 6 | 0.103 | 2.121 | -4.053 | 4.259 | 0.049 | 0.961 |
|  | Mean age | 7 | 0.124 | 0.054 | 0.02 | 0.339 | 2.321 | *0.020* |
|  | Sex | 7 | -0.035 | 0.015 | -0.065 | -0.004 | -2.252 | *0.024* |
|  | Quality of the study | 7 | 0.598 | 0.562 | -0.505 | 1.700 | 1.063 | 0.288 |
| **Quality of life^a,b^** | **DUP** | **4** | **0.038** | **0.026** | **-0.0138** | **0.089** | **1.437** | **0.151** |
|  | % Affective psychosis | 4 | 5.599 | 2.210 | 1.267 | 9.931 | 2.533 | *0.011* |
|  | Mean age | 4 | -0.594 | 0.447 | -1.469 | 0.282 | -1.329 | 0.184 |
|  | Sex | 4 | -0.021 | 0.020 | -0.058 | 0.018 | -1.049 | 0.294 |
|  | Quality of the study | 4 | 0.227 | 0.476 | -0.707 | 1.160 | 0.476 | 0.634 |
| **Functioning** | **DUP** | **7** | **0.002** | **0.003** | **-0.004** | **0.007** | **0.532** | **0.594** |
|  | Continent | 8 | -0.244 | 0.396 | -1.019 | 0.531 | -0.617 | 0.537 |
|  | % Affective psychosis | 7 | 0.009 | 0.992 | -1.934 | 1.953 | 0.010 | 0.992 |
|  | Control content | 8 | -0.352 | 0.218 | -0.779 | 0.075 | -1.615 | 0.106 |
|  | Mean age | 8 | 0.061 | 0.028 | 0.006 | 0.115 | 2.187 | *0.029* |
|  | Sex | 8 | -0.011 | 0.009 | -0.029 | 0.007 | -1.195 | 0.232 |
|  | Quality of the study | 7 | -0.264 | 0.174 | -0.606 | 0.078 | -1.513 | 0.130 |
| **Employment** | **DUP** | **5** | **-0.003** | **0.006** | **-0.014** | **0.008** | **-0.485** | **0.627** |
|  | Continent | 7 | 0.183 | 0.113 | -0.039 | 0.404 | 1.613 | 0.107 |
|  | % Affective psychosis | 6 | -1.185 | 14.374 | -29.358 | 26.799 | -0.082 | 0.934 |
|  | Control content | 7 | -0.298 | 0.154 | -0.600 | 0.004 | -1.932 | 0.053 |
|  | Mean age | 7 | 0.055 | 0.077 | -0.095 | 0.205 | 0.722 | 0.471 |
|  | Sex | 7 | -0.005 | 0.014 | -0.032 | 0.022 | -0.376 | 0.707 |
|  | Quality of the study | 7 | -0.121 | 0.202 | -0.517 | 0.275 | -0.601 | 0.548 |

^a^Control content could not be analysed due to all being TAU.

^b^ Continent could not be analysed due to lack of enough studies for each category.

**eTable 6: Meta-regression analyses between early intervention outcomes and moderating factors**

| **Outcome** |  | **No. of**  **Studies** | **β Coefficient** | **SE** | **95% CI** | | **Z-Value** | **P value** |
| --- | --- | --- | --- | --- | --- | --- | --- | --- |
| **Positive symptoms** | **DUP** | **7** | **-0.067** | **0.085** | **-0.234** | **0.010** | **-0.788** | **0.431** |
|  | Continent | 7 | 0.054 | 0.050 | -0.044 | 0.152 | 1.077 | 0.281 |
|  | % Affective psychosis | 5 | -0.227 | 0.217 | -0.653 | 0.198 | -1.047 | 0.295 |
|  | Control content | 7 | -0.194 | 1.733 | -3.590 | 3.201 | -0.112 | 0.911 |
|  | Mean age | 7 | -0.656 | 1.958 | -4.494 | 3.180 | -0.335 | 0.737 |
|  | Sex | 7 | -0.353 | 1.258 | -2.818 | 2.113 | -0.281 | 0.779 |
|  | Quality of the study | 7 | -0.301 | 0.1667 | -0.628 | 0.026 | -1.804 | 0.071 |
|  | Duration of intervention | 7 | -0.026 | 0.041 | -0.106 | 0.055 | -0.620 | 0.535 |
| **Negative symptoms** | **DUP** | **8** | **0.053** | **0.037** | **-0.019** | **0.126** | **1.436** | **0.151** |
|  | Continent | 8 | -0.117 | 0.364 | -0.830 | 0.596 | -0.321 | 0.748 |
|  | % Affective psychosis | 6 | 1.570 | 1.679 | -1.722 | 4.861 | 0.934 | 0.350 |
|  | Control content | 8 | -0.165 | 0.423 | -0.995 | 0.665 | -0.390 | 0.696 |
|  | Mean age | 8 | -0.057 | 0.04 | -0.137 | 0.022 | -1.418 | 0.156 |
|  | Sex | 8 | -0.003 | 0.018 | -0.038 | 0.033 | -0.150 | 0.881 |
|  | Quality of the study | 8 | 0.300 | 0.176 | -0.044 | 0.644 | 1.709 | 0.087 |
|  | Duration of intervention | 8 | 0.001 | 0.009 | -0.018 | 0.019 | 0.058 | 0.954 |
| **Total psychopathology** | **DUP** | **7** | **0.0044** | **0.018** | **-0.030** | **0.039** | **0.250** | **0.802** |
|  | Continent | 7 | -0.366 | 0.450 | -1.249 | 0.517 | -0.812 | 0.417 |
|  | % Affective psychosis | 6 | 0.994 | 1.076 | -1.115 | 3.103 | 0.924 | 0.355 |
|  | Control content | 7 | 0.299 | 0.180 | -0.054 | 0.651 | 1.660 | 0.097 |
|  | Mean age | 7 | 0.342 | 0.204 | -0.058 | 0.742 | 1.677 | 0.094 |
|  | Sex | 7 | -0.001 | 0.001 | -0.002 | 0.0001 | -1.737 | 0.082 |
|  | Quality of the study | 7 | -0.207 | 0.190 | -0.580 | 0.166 | -1.089 | 0.276 |
|  | Duration of intervention | 7 | -0.002 | 0.003 | -0.008 | 0.004 | -0.593 | 0.553 |
| **Quality of life^a^** | **DUP** | **4** | **0.025** | **0.011** | **0.003** | **0.047** | **2.278** | **0.023** |
|  | Continent | 4 | -0.133 | 0.238 | -0.559 | 0.334 | -0.557 | 0.577 |
|  | % Affective psychosis | 4 | -0.924 | 1.043 | -2.969 | 1.120 | -0.886 | 0.376 |
|  | Mean age | 4 | 0.338 | 0.233 | -0.119 | 0.795 | 1.449 | 0.147 |
|  | Sex | 4 | -0.0002 | 0.017 | -0.034 | 0.034 | -0.013 | 0.989 |
|  | Quality of the study | 4 | 0.076 | 0.321 | -0.554 | 0.706 | 0.237 | 0.812 |
|  | Duration of intervention | 4 | -0.003 | 0.004 | -0.010 | 0.005 | -0.741 | 0.458 |
| **Functioning** | **DUP** | **6** | **0.005** | **0.008** | **-0.011** | **0.021** | **0.628** | **0.530** |
|  | Continent | 6 | 0.014 | 0.238 | -0.453 | 0.480 | 0.059 | 0.953 |
|  | % Affective psychosis | 5 | -1.454 | 2.902 | -7.142 | 4.234 | -0.501 | 0.616 |
|  | Control content | 6 | -0.030 | 0.138 | -0.300 | 0.241 | -0.214 | 0.830 |
|  | Mean age | 6 | 0.011 | 0.029 | -0.045 | 0.068 | 0.410 | 0.682 |
|  | Sex | 6 | -0.009 | 0.009 | -0.026 | 0.008 | -1.023 | 0.306 |
|  | Quality of the study | 6 | 0.096 | 0.119 | -0.138 | 0.330 | 0.803 | 0.422 |
|  | Duration of intervention | 6 | 0.005 | 0.014 | -0.021 | 0.032 | 0.381 | 0.703 |
| **Remission^a^** | **DUP** | **4** | **0.040** | **0.030** | **-0.018** | **0.099** | **1.345** | **0.178** |
|  | Continent | 4 | -0.171 | 0.484 | -1.120 | 0.777 | -0.354 | 0.724 |
|  | % Affective psychosis | 4 | 2.906 | 1.448 | 0.069 | 5.744 | 2.008 | 0.045 |
|  | Mean age | 4 | -0.036 | 0.096 | -0.225 | 0.152 | -0.379 | 0.705 |
|  | Sex | 4 | 0.080 | 0.032 | 0.016 | 0.143 | 2.451 | *0.014* |
|  | Quality of the study | 4 | -0.171 | 0.484 | -1.120 | 0.778 | -0.354 | 0.723 |
|  | Duration of intervention | 4 | -0.005 | 0.005 | -0.015 | 0.006 | -0.866 | 0.386 |
| **Admissions** | **DUP** | **5** | **-0.014** | **0.012** | **-0.037** | **0.009** | **-1.189** | **0.234** |
|  | Continent | 5 | 0.250 | 1.470 | -2.631 | 3.131 | 0.170 | 0.865 |
|  | % Affective psychosis | 5 | -1.262 | 8.026 | -16.993 | 14.470 | -0.157 | 0.875 |
|  | Control content | 5 | -0.547 | 1.418 | -3.325 | 2.231 | -0.386 | 0.670 |
|  | Mean age | 5 | 0.234 | 0.257 | -0.270 | 0.738 | 0.909 | 0.363 |
|  | Sex | 5 | 0.054 | 0.107 | -0.157 | 0.264 | 0.502 | 0.616 |
|  | Quality of the study | 5 | -0.475 | 0.442 | -1.340 | 0.391 | -1.075 | 0.282 |
|  | Duration of intervention | 5 | 0.117 | 0.112 | -0.103 | 0.337 | 1.044 | 0.296 |

^a^Control content could not be analysed due to all being TAU.

**eFigure 1: Forest plots meta-analytic outcomes of early detection strategies**

**Functioning**

Functioning was evaluated with the Global Assessment of Functioning (GAF)^9^, the Social and Occupational Functioning Assessment Scale (SOFAS)^10^ or the Global Functioning: Role (GFR); Global Functioning: Social (GFS)^11,12^.

**Total psychopathology**

Total symptoms were evaluated with the Positive and Negative Syndrome Scale (PANSS)^2^ or the Brief Psychiatric Rating Scale (BPRS)^4^.

**Admission rates**

**Quality of life**

Quality of Life was evaluated with the Quality of Life Scale (QLS)^13^, the Short Form Health Survey (SF-12)^14^ or the World Health Organization Quality of Life (WHO-QoL)^15^

**Positive symptoms**

Positive symptoms were evaluated with the Positive and Negative Syndrome Scale (PANSS)^2^, the Scale for the Assessment of Positive Symptoms (SAPS)^3^ or the Brief Psychiatric Rating Scale (BPRS)^4^.

**Negative symptoms**

Negative symptoms were evaluated with the Positive and Negative Syndrome Scale (PANSS)^2^ or the Scale for the Assessment of Negative Symptoms (SANS)^5^.

**Employment rates**

**Depressive symptoms**

Depressive symptoms were evaluated with the Hamilton Rating Scale for Depression (HAM-D)^6^, the Calgary Depression Scale for Schizophrenia (CDSS)^7^ or the Beck Depression Inventory (BDI)^8^.

**eFigure 2: Forest plots meta-analytic outcomes of early intervention strategies**

**Quality of life**

Quality of Life was evaluated with the Quality of Life Scale (QLS)^13^, the Short Form Health Survey (SF-12)^14^ or the World Health Organization Quality of Life (WHO-QoL)^15^.

**Employment rates**

**Negative symptoms**

Negative symptoms were evaluated with the Positive and Negative Syndrome Scale (PANSS)^2^ or the Scale for the Assessment of Negative Symptoms (SANS)^5^.

**Relapse rates**

**Positive symptoms**

Positive symptoms were evaluated with the Positive and Negative Syndrome Scale (PANSS)^2^, the Scale for the Assessment of Positive Symptoms (SAPS)^3^ or the Brief Psychiatric Rating Scale (BPRS)^4^.

**Remission rates**

**Total psychopathology**

Total psychopathology was evaluated with the Positive and Negative Syndrome Scale (PANSS)^2^ or the Brief Psychiatric Rating Scale (BPRS)^4^.

**Depressive symptoms**

Depressive symptoms were evaluated with the Hamilton Rating Scale for Depression (HAM-D)^6^, the Calgary Depression Scale for Schizophrenia (CDSS)^7^ or the Beck Depression Inventory (BDI)^8^.

**Functioning**

Functioning was evaluated with the Global Assessment of Functioning (GAF)^9^, the Social and Occupational Functioning Assessment Scale (SOFAS)^10^ or the Global Functioning: Role (GFR); Global Functioning: Social (GFS),

**eFigure 3: Funnel plots meta-analytic outcomes of early detection strategies**

**Functioning**

**Total psychopathology**

**Admission rates**

**Quality of life**

**Positive symptoms**

**Negative symptoms**

**Employment rates**

**Depressive symptoms**

**eFigure 4: Funnel plots meta-analytic outcomes of early intervention strategies**

**Quality of life**

**Employment rates**

**Negative symptoms**

**Relapse rates**

**Positive symptoms**

**Remission rates**

**Total psychopathology**

**Depressive symptoms**

**Functioning**

**eFigure 5: Other potential benefits of early detection and early Intervention strategies based on systematic review results**

**eFigure 6: Quality assessment results**

**REFERENCES**

1. Stroup DF, Berlin JA, Morton SC, et al. Meta-analysis of observational studies in epidemiology: a proposal for reporting. Meta-analysis Of Observational Studies in Epidemiology (MOOSE) group. *JAMA.* 2000;283(15):2008-2012.

2. Kay SR, Fiszbein A, Opler LA. The positive and negative syndrome scale (PANSS) for schizophrenia. *Schizophr Bull.* 1987;13(2):261-276.

3. Andreasen N. Scale for the Assessment of Positive Symptoms (SAPS). In. Iowa City: University of Iowa1984.

4. Overall J, Gorham D. The Brief Psychiatric Rating Scale (BPRS): recent developments in ascertainment and scaling. *Psychopharmacol Bull.* 1988;24:97-99.

5. Andreasen N. Scale for the Assessment of Negative Symptoms (SANS). In. Iowa City: University of Iowa1983.

6. Hamilton M. A rating scale for depression. *J Neurol Neurosurg Psychiatry.* 1960;23:56-62.

7. Addington D, Addington J, Maticka-Tyndale E, Joyce J. Reliability and validity of a depression rating scale for schizophrenics. *Schizophr Res.* 1992;6(3):201-208.

8. Beck A, Steer R, Brown G. Manual for the beck depression inventory-II. In. Psychological Corporation, San Antonio, TX1996.

9. Piersma HL, Boes JL. The GAF and psychiatric outcome: a descriptive report. *Community Ment Health J.* 1997;33(1):35-41.

10. Morosini PL, Magliano L, Brambilla L, Ugolini S, Pioli R. Development, reliability and acceptability of a new version of the DSM-IV Social and Occupational Functioning Assessment Scale (SOFAS) to assess routine social functioning. *Acta Psychiatr Scand.* 2000;101(4):323-329.

11. Niendam TA, Bearden CE, Johnson JK, et al. Neurocognitive performance and functional disability in the psychosis prodrome. *Schizophrenia Research.* 2006;84(1):100-111.

12. Cornblatt BA, Auther AM, Niendam T, et al. Preliminary findings for two new measures of social and role functioning in the prodromal phase of schizophrenia. *Schizophrenia Bulletin.* 2007;33(3):688-702.

13. Heinrichs DW, Hanlon TE, Carpenter WT. The Quality of Life Scale: an instrument for rating the schizophrenic deficit syndrome. *Schizophr Bull.* 1984;10(3):388-398.

14. Ware J, Kosinski M, Keller SD. A 12-Item Short-Form Health Survey: construction of scales and preliminary tests of reliability and validity. *Med Care.* 1996;34(3):220-233.

15. WHOQOL Group. Development of the World Health Organization WHOQOL-BREF quality of life assessment. *Psychol Med.* 1998;28(3):551-558.

16. Correll CU, Galling B, Pawar A, et al. Comparison of Early Intervention Services vs Treatment as Usual for Early-Phase Psychosis: A Systematic Review, Meta-analysis, and Meta-regression. *JAMA Psychiatry.* 2018;75(6):555-565.

17. Intensive Early Intervention Programme for Incipient Psychosis (PAE‐TPI ) vs. Standard Treatment (ST) Implemented in the Public Mental Health Network of Catalonia: accessibility measures. Early Intervention in Psychiatry. Australia: John Wiley & Sons Australia, Ltd; 2016.

18. Bertelsen M, Jeppesen P, Petersen L, et al. Five-year follow-up of a randomized multicenter trial of intensive early intervention vs standard treatment for patients with a first episode of psychotic illness. *Archives of General Psychiatry.* 2008;65(7):762-771.

19. Browne J, Penn DL, Bauer DJ, et al. Perceived Autonomy Support in the NIMH RAISE Early Treatment Program. *Psychiatric Services.* 2017;68(9):916-922.

20. Cassidy CM, Schmitz N, Norman R, Manchanda R, Malla A. Long-term effects of a community intervention for early identification of first-episode psychosis. *Acta Psychiatrica Scandinavica.* 2008;117(6):440-448.

21. Chan SKW, So HC, Hui CLM, et al. 10-year outcome study of an early intervention program for psychosis compared with standard care service. *Psychological Medicine.* 2015;45(6):1181-1193.

22. Chan SKW, Chau EHS, Hui CLM, Chang WC, Lee EHM, Chen EYH. Long term effect of early intervention service on duration of untreated psychosis in youth and adult population in Hong Kong. *Early Intervention in Psychiatry.* 2018;12(3):331-338.

23. Chan SKW, Chan SWY, Pang HH, et al. Association of an Early Intervention Service for Psychosis With Suicide Rate Among Patients With First-Episode Schizophrenia-Spectrum Disorders. *Jama Psychiatry.* 2018;75(5):458-464.

24. Chong SA, Ravichandran N, Poon LY, Soo KL, Verma S. Reducing polypharmacy through the introduction of a treatment algorithm: Use of a treatment algorithm on the impact on polypharmacy. *Annals Academy of Medicine Singapore.* 2006;35(7):457-460.

25. Chong SA, Mythily S, Verma S. Reducing the duration of untreated psychosis and changing help-seeking behaviour in Singapore. *Social Psychiatry and Psychiatric Epidemiology.* 2005;40(8):619-621.

26. Connor C, Birchwood M, Freemantle N, et al. Don't turn your back on the symptoms of psychosis: the results of a proof-of-principle, quasi-experimental intervention to reduce duration of untreated psychosis. *Bmc Psychiatry.* 2016;16.

27. Craig TK, Garety P, Power P, et al. The Lambeth Early Onset (LEO) Team: randomised controlled trial of the effectiveness of specialised care for early psychosis. *BMJ.* 2004;329(7474):1067.

28. Cullberg J, Levander S, Holmqvist R, Mattsson M, Wieselgren IM. One-year outcome in first episode psychosis patients in the Swedish Parachute project. *Acta Psychiatrica Scandinavica.* 2002;106(4):276-285.

29. Ferrara M, Guloksuz S, Li F, et al. Parsing the impact of early detection on duration of untreated psychosis (DUP): Applying quantile regression to data from the Scandinavian TIPS study. *Schizophr Res.* 2019;210:128-134.

30. Hegelstad WT, Larsen TK, Auestad B, et al. Long-Term Follow-Up of the TIPS Early Detection in Psychosis Study: Effects on 10-Year Outcome. *American Journal of Psychiatry.* 2012;169(4):374-380.

31. Joa I, Johannessen JO, Auestad B, et al. The key to reducing duration of untreated first psychosis: Information campaigns. *Schizophrenia Bulletin.* 2008;34(3):466-472.

32. Kane JM, Robinson DG, Schooler NR, et al. Comprehensive Versus Usual Community Care for First-Episode Psychosis: 2-Year Outcomes From the NIMH RAISE Early Treatment Program. *American Journal of Psychiatry.* 2016;173(4):362-372.

33. Keating D, McWilliams S, Boland F, et al. Prescribing pattern of antipsychotic medication for first-episode psychosis: a retrospective cohort study. *BMJ Open.* 2021;11(1):e040387.

34. Krstev H, Carbone S, Harrigan SM, Curry C, Elkins K, McGorry PD. Early intervention in first-episode psychosis - The impact of a community development campaign. *Social Psychiatry and Psychiatric Epidemiology.* 2004;39(9):711-719.

35. Lambert M, Schottle D, Ruppelt F, et al. Early detection and integrated care for adolescents and young adults with psychotic disorders: the ACCESS III study. *Acta Psychiatrica Scandinavica.* 2017;136(2):188-200.

36. Larsen TK, Melle I, Friis S, et al. One-year effect of changing duration of untreated psychosis in a single catchment area. *British Journal of Psychiatry.* 2007;191:S128-S132.

37. Lloyd-Evans B, Sweeney A, Hinton M, et al. Evaluation of a community awareness programme to reduce delays in referrals to early intervention services and enhance early detection of psychosis. *Bmc Psychiatry.* 2015;15.

38. Malla A, Jordan G, Joober R, et al. A controlled evaluation of a targeted early case detection intervention for reducing delay in treatment of first episode psychosis. *Soc Psychiatry Psychiatr Epidemiol.* 2014;49(11):1711-1718.

39. Malla A, Norman R, Scholten D, Manchanda R, McLean T. A community intervention for early identification of First Episode Psychosis - Impact on duration of untreated psychosis (DUP) and patient characteristics. *Social Psychiatry and Psychiatric Epidemiology.* 2005;40(5):337-344.

40. McGorry PD, Edwards J, Mihalopoulos C, Harrigan SM, Jackson HJ. EPPIC: An evolving system of early detection and optimal management. *Schizophrenia Bulletin.* 1996;22(2):305-326.

41. Melle I, Larsen TK, Haahr U, et al. Reducing the duration of untreated first-episode psychosis - Effects on clinical presentation. *Archives of General Psychiatry.* 2004;61(2):143-150.

42. Melle I, Larsen TK, Haahr U, et al. Prevention of negative symptom psychopathologies in first-episode schizophrenia. *Archives of General Psychiatry.* 2008;65(6):634-640.

43. Melle I, Johannessen JO, Friis S, et al. Course and Predictors of Suicidality Over the First Two Years of Treatment in First-Episode Schizophrenia Spectrum Psychosis. *Archives of Suicide Research.* 2010;14(2):158-170.

44. Mihalopoulos C, Harris M, Henry L, Harrigan S, McGorry P. Is Early Intervention in Psychosis Cost-Effective Over the Long Term? *Schizophrenia Bulletin.* 2009;35(5):909-918.

45. Nishida A, Ando S, Yamasaki S, et al. A randomized controlled trial of comprehensive early intervention care in patients with first-episode psychosis in Japan: 1.5-year outcomes from the J-CAP study. *Journal of Psychiatric Research.* 2018;102:136-141.

46. Petrakis M, Penno S, Oxley J, Bloom H, Castle D. Early psychosis treatment in an integrated model within an adult mental health service. *European Psychiatry.* 2012;27(7):483-488.

47. Srihari VH, Tek C, Kucukgoncu S, et al. First-Episode Services for Psychotic Disorders in the US Public Sector: A Pragmatic Randomized Controlled Trial. *Psychiatric Services.* 2015;66(7):705-712.

48. Srihari V, Guloksuz S, Li F, et al. Mindmap: a population-based approach to early detection of psychosis in the United States. Paper presented at: International Congress on Schizophrenia Research2017.

49. Valmaggia LR, Byrne M, Day F, et al. Duration of untreated psychosis and need for admission in patients who engage with mental health services in the prodromal phase. *British Journal of Psychiatry.* 2015;207(2):130-134.
